# Cortical and Spinal Mechanisms of Task Failure of Sustained Submaximal Fatiguing Contractions

**DOI:** 10.1371/journal.pone.0093284

**Published:** 2014-03-25

**Authors:** Petra S. Williams, Richard L. Hoffman, Brian C. Clark

**Affiliations:** 1 Ohio Musculoskeletal & Neurological Institute (OMNI), Ohio University, Athens, Ohio, United States of America; 2 Department of Physical Therapy and Athletic Training, Northern Arizona University, Flagstaff, Arizona, United States of America; 3 Department of Biomedical Sciences, Ohio University, Athens, Ohio, United States of America; 4 Department of Geriatric Medicine and Gerontology, Ohio University, Athens, Ohio, United States of America; The University of Queensland, Australia

## Abstract

In this and the subsequent companion paper, results are presented that collectively seek to delineate the contribution that supraspinal circuits have in determining the time to task failure (TTF) of sustained submaximal contractions. The purpose of this study was to compare adjustments in supraspinal and spinal excitability taken concurrently throughout the performance of two different fatigue tasks with identical mechanical demands but different TTF (i.e., force-matching and position-matching tasks). On separate visits, ten healthy volunteers performed the force-matching or position-matching task at 15% of maximum strength with the elbow flexors to task failure. Single-pulse transcranial magnetic stimulation (TMS), paired-pulse TMS, paired cortico-cervicomedullary stimulation, and brachial plexus electrical stimulation were delivered in a 6-stimuli sequence at baseline and every 2–3 minutes throughout fatigue-task performance. Contrary to expectations, the force-matching task TTF was 42% shorter (17.5±7.9 min) than the position-matching task (26.9±15.11 min; p<0.01); however, both tasks caused the same amount of muscle fatigue (*p* = 0.59). There were no task-specific differences for the total amount or rate of change in the neurophysiologic outcome variables over time (p>0.05). Therefore, failure occurred after a similar mean decline in motorneuron excitability developed (*p*<0.02, ES = 0.35–0.52) coupled with a similar mean increase in measures of corticospinal excitability (*p*<0.03, ES = 0.30–0.41). Additionally, the amount of intracortical inhibition decreased (*p*<0.03, ES = 0.32) and the amount of intracortical facilitation (*p*>0.10) and an index of upstream excitation of the motor cortex remained constant (*p*>0.40). Together, these results suggest that as fatigue develops prior to task failure, the increase in corticospinal excitability observed in relationship to the decrease in spinal excitability results from a combination of decreasing intracortical inhibition with constant levels of intracortical facilitation and upstream excitability that together eventually fail to provide the input to the motor cortex necessary for descending drive to overcome the spinal cord resistance, thereby contributing to task failure.

## Introduction

For healthy individuals performing sustained contractions, fatigue is an expected and normal physiologic reaction that inevitably leads to task failure [1], [2]. Muscle fatigue is defined as the gradual decline in maximum muscle force capacity relative to pre-fatigue values and can be viewed as the development of activity dependent weakness that resolves with rest [1], [3], [4]. During sustained submaximal contractions, muscle fatigue will be present *prior* to task failure; however, task performance will continue for a period of time without appreciable disruption [1], [3], [5]–[7]. As task duration increases, muscle fatigue will progress to a degree such that it interferes with the capacity to sustain the precise amount of submaximal force output required (e.g. force output fluctuates around the target force) impeding accuracy of performance and eventually prohibiting effective task performance. Task failure becomes the point in time when the force output required for successful task performance can no longer be sustained as demanded by the activity [5].

It is well accepted that there is not one single cause of fatigue; instead, the physiologic mechanisms behind the decline in force output that prohibits indefinite task performance are specific to the task demands (i.e., contraction intensity, duration, mode, muscle group, joint angle, limb posture, and stabilization) that collectively stress different regions of the neuromuscular pathway responsible for the contraction in order to sustain the required force output [1], [3], [4], [7]–[12]. In general, it has been shown that the nervous system’s failure to maintain sufficient activation of the muscle is a significant contributor to task failure in sustained submaximal contractions as compared to maximal contractions [1], [5], [6], [8], [13], [14]. Additionally, under otherwise identical mechanical demands during sustained submaximal contractions, neurophysiologic measures have also been found to differ with load compliance (e.g. exert a constant force against a rigid restraint or hold an equivalent inertial weight) which requires individuals to attend to distinct performance feedback variables, to either force output (*force-matching task*) or joint angle position (*position-matching task*) [5], [7], [12], [15], [16]. Typically, submaximal contractions performed with the elbow flexors at an intensity up to 30% of maximum force result in the same amount of muscle fatigue at task failure; however, the time to task failure (TTF) is nearly twice as long for the force-matching task compared to the position-matching task [12], [15], [16].

The excitatory drive to the muscle by the motorneurons during sustained submaximal contractions can be modulated by supraspinal inputs from descending pathways, spinal inputs from interneurons and peripheral afferents, and also by intrinsic changes of the motorneuron itself [1], [7], [17]–[19]. Evoked potentials elicited by transcranial magnetic stimulation (TMS) of the motor cortex and electrical stimulation of the spinal tracts or the peripheral nerve during task performance provide evidence about the state of nervous system excitability and have been suggested to be an indirect assessment of the amount of nervous system activation [20], [21]. It is important to recognize that this experimental strategy does not directly assess the amount of activation provided to the motor pool from the motor cortex or spinal afferents rather it examines the responsivity of the nervous system to external stimulation at the time of stimulation from which interpretations about the segmental state of activity are made [21]. Inferences about the state of activation in a region relative to another are more plausible when cortical, spinal and peripheral measures are evoked concurrently, in real-time and also during different states of activation (i.e., fatigued vs. non-fatigued, resting vs. active) [22]–[24].

The amplitude of a motor evoked potential (MEP) evoked by a single suprathreshold TMS pulse to the motor cortex provides a composite index of excitability of the entire voluntary motor pathway, as the size of the response depends upon both cortical and spinal excitability [1], [25], [26]. During sustained submaximal contractions, much like the voluntary electromyographic (EMG) signal, MEP amplitude has been shown to increase as task duration increases [1], [22], [27], [28]. This increase in responsivity to the external stimulus has been attributed to enhanced voluntary drive both to and from the motor cortex and to increased descending drive to recruit motorneurons in order to sustain neural output and thus muscle activation [21]–[23], [28], [29]. These findings are consistent with evidence from single motor unit studies that report progressive recruitment of new, unfatigued motor units to sustain force output that underlies the increase in voluntary EMG amplitude throughout task performance [17], [30], [31].

When evoked during a voluntary contraction, the MEP is followed by an electrical silent period (SP), observed as a transient cessation of ongoing EMG activity consistent with an interruption in volitional drive to the cortex and withdrawal of descending input to the spinal motorpool [32]. In non-fatiguing contractions, the SP duration has been attributed to an initial short period of spinal refractoriness that recovers (∼50msec) combined with a longer period of cortical inhibition (up to ∼200 msec) [29], [32]. During fatiguing contractions, the SP duration also increases, and this increase has been assumed to be due to the same mechanism of enhanced cortical inhibition reported for a longer SP with non-fatiguing contractions [1], [15], [33], [34]. This interpretation of increased SP duration with fatiguing contractions has posed a conundrum in that despite an increase in cortical excitability as measured by the increased in MEP amplitudes, there also appears to be a concurrent increase in cortical inhibition [1], [15], [35]. This raises the question as to the segmental contribution of intracortical circuits within the motor cortex, and also of “upstream” inputs to the motor cortex, to task failure during sustained submaximal contractions [1], [15], [22], [34], [36]. In other words, during task performance does active cortical inhibition develop or is there insufficient intracortical facilitation and “upstream input” that limits the capacity of supraspinal structures to sustain sufficient excitatory drive to the motorneuron pool and thus contribute to task failure?

Paired-pulse TMS protocols, including short-interval intracortical inhibition (SICI) and intracortical facilitation (ICF), provide a strategy to more directly evaluate the excitability of intracortical interneuron networks within the motor cortex [20], [37]–[39]. The effect of a subthreshold conditioning pulse that activates the cortical interneurons on the MEP amplitude of a subsequent suprathreshold test pulse is compared relative to a single MEP. When separated by interstimulus intervals (ISI) of 2–5msec, the evoked MEP amplitude decreases and the SICI ratio of the test MEP to the single MEP is less than 1.0 reflecting intracortical inhibition [38], [40]. An ISI of 12–25msec increases the test MEP amplitude and generally, the ratio is greater than 1.0, which is interpreted as representing ICF [20], [37]. These two measures of intracortical excitability have rarely been used to monitor the ongoing adjustments in intracortical excitability related to fatigue [41]–[43] and, to date, have not been employed to examine intracortical networks during the performance of submaximal contractions sustained to task failure.

Electrical stimulation to the spinal cord tracts at the cervicomedullary junction transynaptically activates the motor neuron pool and elicits a cervicomedullary evoked potential (CMEP) regarded as a segmental index of spinal alpha-motorneuron excitability [44], [45]. The CMEP response latency suggests that the CMEP reflects a monosynaptic relationship between the corticospinal tract and the motorneuron without influence from pre-synaptic inhibition of the corticospinal tract [45], [46]. Collision experiments have demonstrated that MEPs and CMEPs are transmitted in the same axons of the corticospinal tract; therefore, it is practical to compare the two responses to differentiate cortical relative to spinal motorneuron adaptations as fatigue develops [44]–[46]. A technique introduced by McNeil et al., where MEPs and CMEPs are evoked during the SP (i.e., stimulating during the period of electrical silence after a single suprathreshold TMS pulse) provides a strategy to assess the excitability of the corticospinal system and the motorneurons independent of upstream voluntary drive to the cortex and descending drive during a fatiguing contraction [22], [23], [47].

In the non-fatigued state, the amplitudes of a MEP or a CMEP evoked in the SP are less than those evoked in the presence of voluntary activation such that a ratio of the SP evoked response to the single control pulse is typically <1.0 [22], [47]. In addition, the MEP ratio is also less than the CMEP ratio suggesting the added presence of intracortical inhibition in the non-fatigued state [22], [47]. The ratio of the MEP evoked in the SP to the single MEP is referred to as long-interval intracortical inhibition (LICI) and is thought to be influenced by cortical inhibition mechanisms similar to those influencing the SP duration in the non-fatigued state [1], [24]. The cortically evoked MEP amplitude depends upon composite cortical and spinal excitability–whether or not it is evoked in the SP–and recent work by McNeil et al., suggests that the term long-interval inhibition (LII) may be a more accurate label for this ratio as opposed to LICI [22], [23], [47], [48].

During the performance of sustained submaximal contractions where subjects were asked to maintain a consistent level of voluntary EMG activity (as opposed to force output), the amplitudes of the CMEP evoked in the SP and the MEP evoked in the SP declined in parallel to each other and to values well-below pre-fatigue measures [23]. McNeil and colleagues concluded that because the values for the cortical and spinal measures declined in parallel, as opposed to seeing a greater decline in the cortically evoked MEPs, the motor cortex is not actively inhibited during sustained submaximal contractions. Instead, they propose that the motorneurons become progressively resistant to stimulation as the task progresses which suggests that changes in LII as well as in SP duration are more likely due to decreased motorneuron excitability rather than to increased cortical inhibition when measured in the fatigued state [22], [23], [47]–[49].

Studies examining the functional significance of neurophysiologic adjustments to the difference in TTF between the force-matching and the position-matching tasks have provided valuable insight into the spinal mechanisms that limit the duration of sustained submaximal contractions. The shorter TTF for the position-matching task has been associated with a greater reduction in H-reflex amplitude (a global index of spinal excitability) [27], [50] and no decline in 1a pre-synaptic inhibition measured by the conditioned H-reflex (an indirect index of pre-synaptic inhibition) when compared to the force-matching task (where 1a pre-synaptic inhibition declines) [17]. To date, only one study has investigated the differences in supraspinal excitability between the two tasks using single pulse TMS [27]. Non-significant differences were observed in the rates of increase in MEP amplitude and EMG amplitude; however, at task failure the longer duration force-matching task had greater MEP and EMG amplitude and a longer SP duration while the shorter duration position-matching task had greater rate and amount of reduction in H-reflex amplitude. Together these results suggest insufficient motor unit recruitment contributed to early position-matching task failure [7], [15], [27]. While inconclusive regarding supraspinal mechanisms the results from this study, when combined with prior reports from single motor unit studies about recruitment threshold and discharge rates, have been interpreted to suggest that the shorter TTF for the position-matching is associated with decreased facilitation of the motorneuron pool from peripheral afferents due to sustained or even increased pre-synaptic inhibition of the Ia afferent by descending inputs in order to manage force fluctuations as opposed to intrinsic changes to the motorneuron itself [5], [15].

The neurophysiologic studies reviewed here have provided convincing evidence to support the conclusion that early task failure is associated with a faster rate of motor unit recruitment by descending inputs in order to compensate for a rapid decline in spinal excitability [12], [17], [22], [23], [27], [29], [31]; nevertheless, these findings do not rule out the potential that supraspinal mechanisms influencing descending drive also contribute to task failure as despite the compensation, task failure remains inevitable. Studies that have employed single pulse TMS suggest that there is a simultaneous increase in both cortical excitability (i.e., increase MEP amplitude) and inhibition (i.e., increase SP duration) [1], [15], [22], [33]–[36]. Therefore, the purpose of this study was to delineate supraspinal adjustments, including intracortical facilatory and inhibitory circuits and voluntary drive “upstream” of the motor cortex, relative to concurrent spinal adjustments in excitability to determine the functional significance on the difference in TTF for the force-matching and position-matching tasks. We used a combination of single-pulse TMS, paired-pulse TMS and paired cortico-cervicomedullary stimulation as alone these measures do not directly quantify the amount of neural activation; however, if interpreted relative to the others greater inference to the state of nervous system activity is possible [20], [21], [51]. We hypothesized that the shorter TTF for the position-matching task would be associated with a greater rate of reduction in alpha-motorneuron excitability and a greater rate of increase in the measures cortical excitability, without an associated increase in measures of intra-cortical inhibition.

## Methods

### Subjects

Ten healthy, right-handed individuals volunteered to participate in this study (5 men, 5 women; 24.5±3.10 yrs, 174.5±12.5 cm, 75.73±20.92 kg). Prior to participation, each subject attended an orientation session where they completed a series of questionnaires to confirm they were free from any known contraindications to either stimulation (magnetic or electrical) or exercise due to a neurologic disorder, cardiovascular disease, or musculoskeletal injury in the upper extremities. Subjects identified themselves as highly active (n = 2, 1 male, 1 female,) moderately active (n = 5, 3 male, 2 female), or low active (n = 3, 1 male, 2 female) based on the Lipid Research Clinics Physical Activity Questionnaire [52], but denied participating in resistance training in the prior 3-months. Handedness was evaluated using the Edinburgh Handedness Inventory (mean score: 74±19%) with scores greater than 40% indicating right hand dominance [53]. During the orientation, subjects were familiarized to the neurophysiologic testing methods and the experimental procedures, but they remained naïve to the prior research on differences in TTF for the two tasks.

### Ethics Statement

The Institutional Review Board at Ohio University approved the study protocol, and all study participants provided written informed consent.

### General Overview of the Experiment and Testing Sessions

Subjects participated in two experimental sessions separated by 5–8 days (mean days separating sessions: 6.5±1.1 days) conducted at the same time of day for each subject. Subjects were randomly assigned to a counterbalanced order of fatigue-task conditions (Force-matching task visit 1∶2 men and 3 women; Position-matching task visit 1∶3 men and 2 women). During each testing session, subjects performed one of two sustained submaximal fatiguing contraction tasks with the elbow flexors of the non-dominant arm at an intensity equal to 15% of maximal voluntary contraction (MVC) until volitional task failure. The two fatigue tasks followed an identical protocol and had identical mechanical demands, including identical net muscle force, joint angle, limb posture and stabilization, but differed in load compliance (i.e., force-matching vs. position-matching task). The difference in load compliance required the subjects to attend to distinct performance feedback variables during each task; either the force output from the elbow flexors (*force-matching task*) or the joint angle position of the elbow joint (*position-matching task*). During the force-matching task, subjects were asked to sustain a consistent 15% MVC force output for as long and as accurately as possible as they pulled against a force transducer tethered to the chair whose length, when taut, prevented the elbow from flexing more than 90°. For the position-matching task, subjects supported a free-hanging, untethered weight equivalent to 15% MVC force and focused on maintaining the elbow joint position at 90° as long and as precisely as possible until task failure. Subjects received task-specific visual feedback about either the force output or joint position throughout task performance. Muscle activation patterns of the biceps brachii and brachioradialis muscles were examined during the respective tasks by quantifying the amplitude of the interference electromyogram signals. Adjustments in cortical, spinal, and muscle excitability during task performance were assessed prior to and during the fatigue tasks. Here, a sequence of 6 electrical and magnetic stimuli were delivered to the motor cortex (TMS), the cervicomedullary junction, and the brachial plexus at regular intervals. The performance outcomes were TTF, and muscle fatigue quantified as the reduction in MVC force immediately following the fatigue task. Eight neurophysiologic outcome measures were quantified from the evoked responses: 1) MEP amplitude, 2) SP duration, 3) MEP amplitude elicited during the corticospinal SP (MEP in SP), 4) paired-pulse MEP ratio of long interval inhibition (LII), 5) paired-pulse MEP ratio of SICI, 6) paired-pulse MEP ratio of ICF, 7) CMEP’s elicited during the SP (CMEP in SP; as an index of motorneuron excitability independent from descending drive), and 8) M_max_ (used as an index of muscle excitability and to permit normalization of all of the neurophysiologic amplitude evoked potential measures). To permit analysis of changes in the neurophysiologic outcome variables between fatigue-tasks and relative to each other during the fatigue-tasks, the outcome variables were normalized and expressed as a percentage of the baseline pre-fatigue value.

**Figure 1:**
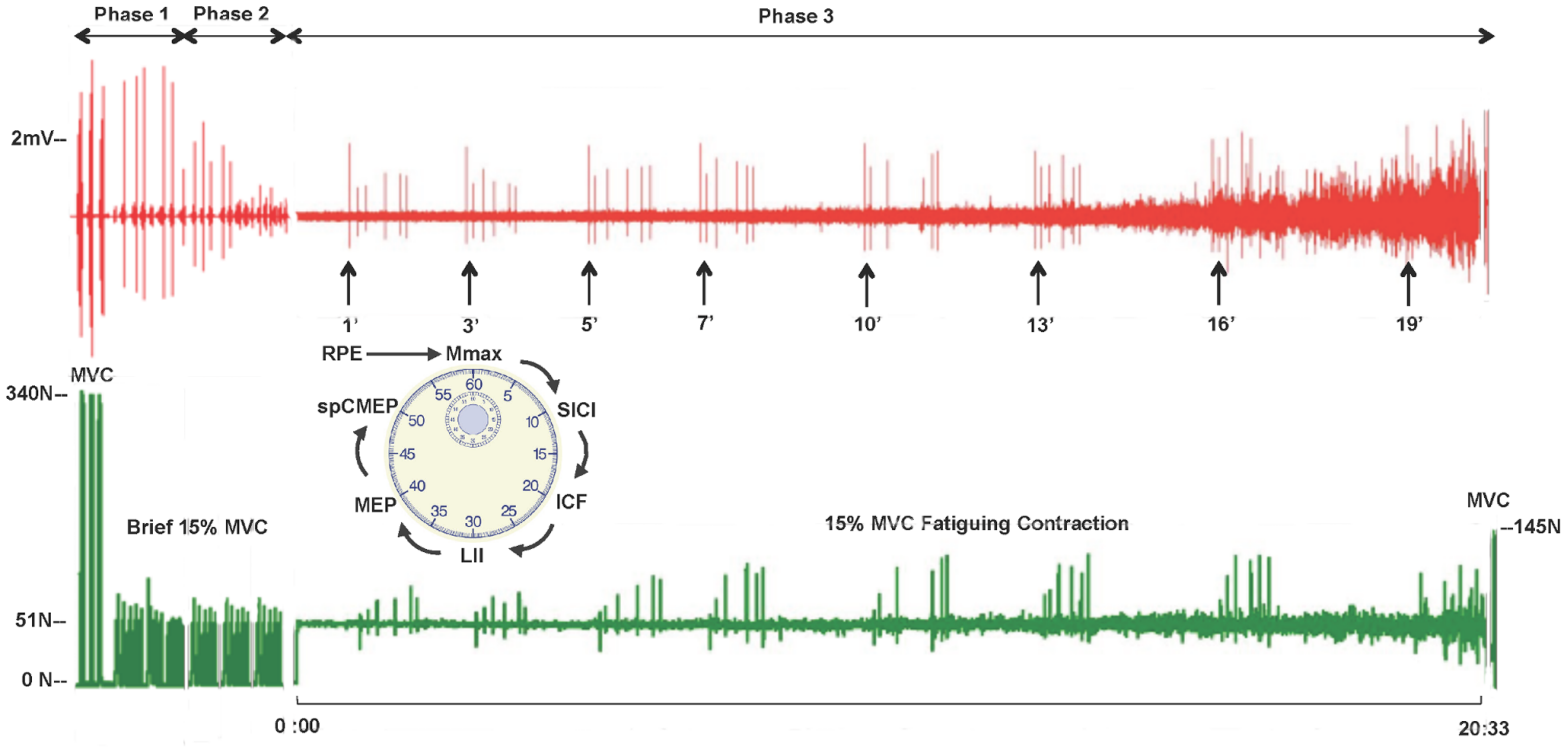
Experimental protocol. *(Top)* The three phases of the testing protocol. Phase 1 involved strength testing and stimulus intensity determination for evoked signals; in Phase 2 pre-fatigue baseline measures for evoked signals were taken 3 times; and in Phase 3 subjects performed the sustained, submaximal fatigue task during which the stimulus protocol was delivered at predetermined intervals. At task failure subjects performed a final MVC. *(Middle)* Representative trace of the biceps electromyogram (EMG) and time points when this subject received the stimulus protocol (see clock insert for sequence delivered over 60 seconds). *(Bottom)* Representative trace of the recorded force signal during the force-matching task. This subject’s time to task failure was 20∶30 minutes. *ICF: intracortical facilitation; LII: long interval inhibition; MEP: motor evoked potential; Mmax: maximum compound muscle action potential; MVC: maximum voluntary contraction; N: newtons; RPE: rating of perceived exertion; SICI: short interval intracortical inhibition; spCMEP: cervicomedullary evoked potential elicited in the silent period.*

The experimental protocol for both testing sessions was identical and consisted of three phases (**Figure 1**). Subjects were positioned in a custom-made chair, fit with a wrist orthosis, and prepped for EMG recordings. Phase 1 started by testing the elbow flexor MVC to measure pre-fatigue strength and determine the 15% MVC target force to be sustained during the fatigue tasks. Subjects then performed several 15% MVC short-duration (3–5 sec) force-matching contractions during which the stimulation intensities for TMS, cervicomedullary, and peripheral nerve stimulation were determined. The values for the target force and the stimulus intensities identified in test session 1 were confirmed during test session 2, and used for both test sessions. During phase 2, a total of 3 pre-fatigue baseline values for each neurophysiologic outcome variable were obtained. At the conclusion of the baseline testing, subjects were given a break before starting the fatigue task. For phase 3, subjects performed either the force-matching or the position-matching fatigue task to task failure. One-minute into the fatigue task, subjects were asked their rating of perceived exertion (RPE) using the Modified Borg 0–10 scale [54], which was immediately followed by a 60-sec sequence of 6 total magnetic and electrical stimulation pulses (1 every 10-secs). The RPE/stimulation measures were repeated every 2-min up to 7 minutes (i.e., 1, 3, 5, and 7-mins), after which they were taken every 3-min (e.g. 10, 13, 16-min, etc.) until task failure. Subjects were not informed of the timing of stimulation or their contraction times. A final RPE was taken at task failure. Five seconds after task failure, subjects performed one last MVC. Subjects were only told their TTF after completing both test sessions.

### Experimental Setup and Mechanical Recordings

**Figure 2:**
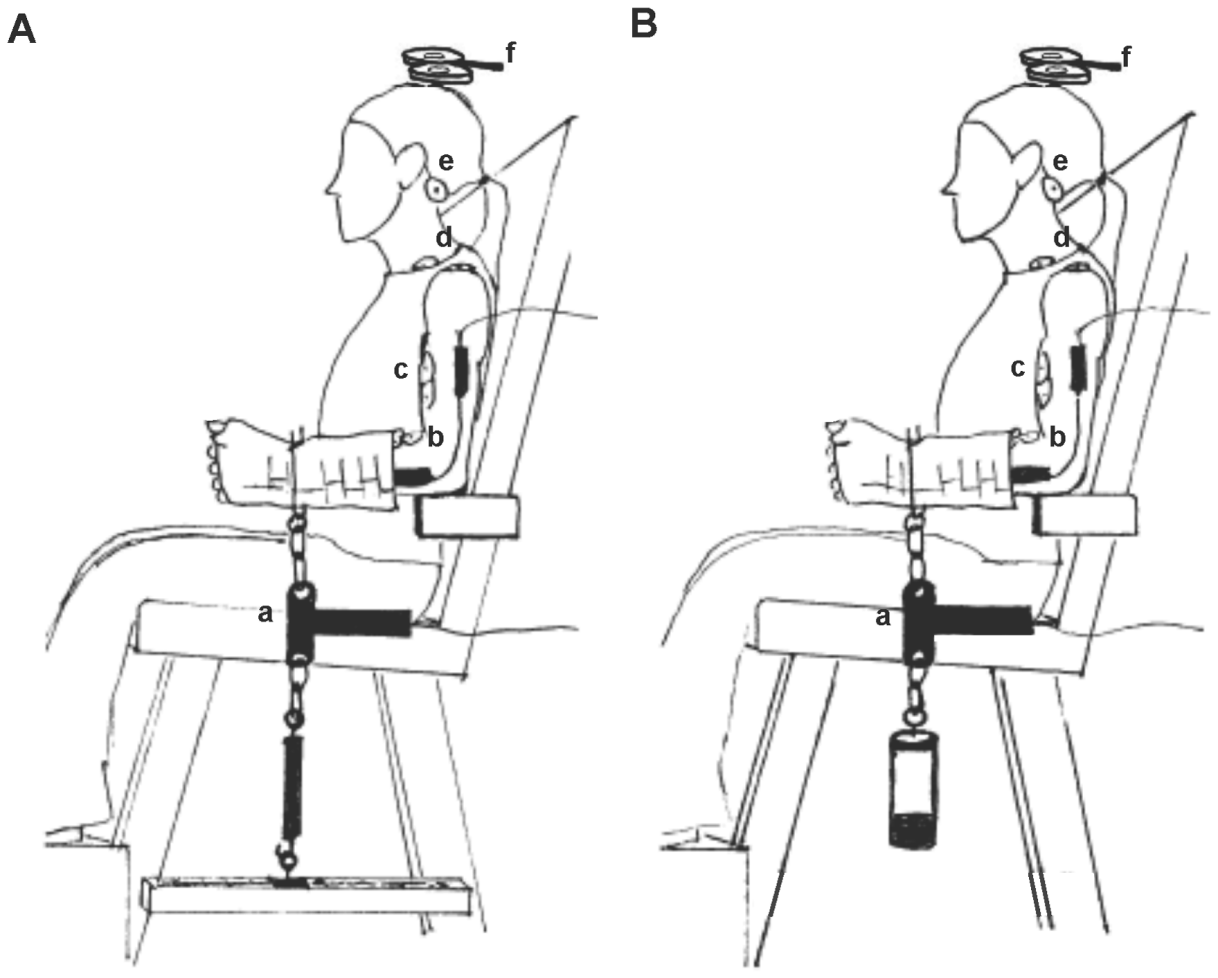
Experimental setup and subject positioning for the force-matching task (A) and the position-matching task (B). Special care was taken to ensure that the mechanical demands of each task were identical, and no external restraints were used to restrict motions of the torso, shoulder, elbow, or forearm in either test session. A. During the force-matching task the orthosis was anchored to the chair base via an adjustable length tether that became taut with the elbow flexed to 90°. The force transducer (a), placed in series between the anchor and the orthosis, measured the amount of force exerted by the elbow flexors through the tether against the anchor. The force output (15% MVC) was displayed on a computer monitor to provide visual feedback. The elbow joint angle was determined and confirmed by an electrogoniometer (b), electromyographic signals were recorded from the biceps brachii and brachioradialis muscles (c), electrical stimulation was delivered at Erb’s point (d) and the cervicomedullary junction (e), and transcranial magnetic stimulation was delivered to the motor cortex (f). B. During the position-matching fatigue-task the magnitude of the suspended weight was equivalent to 15% MVC confirmed by the force transducer (a), and the elbow joint angle, measured via an electrogoniometer, was displayed on a computer monitor to provide visual feedback (b). An identical electromyographic (c), electrical stimulation (d and e), and transcranial magnetic stimulation (f) setup as described for the force-matching task was also implemented for the position-matching task.

To provide consistent mechanical demands between the two tasks, the upper limb position, proximal segment stabilization, and joint torque (force×moment arm) were identical. Subjects were seated in an upright adjustable chair with the left arm positioned next to the body in 10–15° of humeral abduction which placed the olecranon process of the elbow joint on the small padded rest used to support the weight of the upper arm. This shoulder joint alignment has been shown to minimize stress on the rotator cuff muscles during fatigue-task performance [55]. The elbow rest did not restrict motions of the humerus or the forearm. With the torso resting against the back of the chair and the shoulder joint aligned with 0° flexion/extension, the humerus was vertical and the forearm parallel to the ground to position the elbow joint at 90°. The forearm was in neutral rotation with the thumb pointed towards the ceiling. The shoulder joint was oriented in neutral rotation so that when the elbow flexed, the thumb aimed towards the acromion process of the shoulder, not the subject’s chin. To obviate the use of the hand and wrist muscles during testing as well as to provide a secure attachment for the loads, the forearm and hand were immobilized in a pre-fabricated Wrist-Hand-Thumb-Orthosis (Model 100, Orthomerica, Newport Beach, CA). This composite arm posture has been found to provide a consistently significant difference in TTF between the force-matching and position-matching tasks with the elbow flexors for loads below 45% MVC for both men and women between ages 18 and 45 years [16], [17], [56], [57]. A 14-inch computer monitor that provided visual feedback about task performance was placed 1-meter in front of the subject and the height aligned at eye level to ensure that subjects would not alter their sitting posture (and therefore their arm position) in order to view the screen [58]. It is important to note that external supports (i.e. straps) were not used to restrict motions at the shoulder or the torso in order to equalize the demand placed on synergists, proximal joints, and postural stabilizers between the two fatigue-tasks. A schematic illustration of the experimental setup is shown in **Figure 2**.

The loads were attached on the ulnar side of the wrist to the bend of a “U” shaped bolt that was secured around the orthosis just proximal to the wrist joint. This permitted the load to slide unrestricted in the frontal plane thus uncoupling forearm pronation and supination motions from elbow joint flexion angle. For the MVC tests and the 15% MVC force-matching task, the orthosis was tethered to an anchor point on the chair base. The length of the tether was set so that when it was pulled taut by the vertically directed force of the contracting elbow flexors, the elbow joint was flexed to 90° with the forearm parallel to the ground. Unlike in other studies where the orthosis was clamped into place, restricting forearm motion in all planes [16], [17], [31], [57], [59], [60], the tether only prevented further elbow flexion beyond 90°; no other motions in the arm were restricted. During the position-matching task, the total weight suspended from the orthosis was equivalent to 15%MVC. To ensure consistent torque demands between tasks, the moment arm length for the force was the measured distance between the “U” bolt and the posterior elbow on the padded rest and the forearm parallel to the ground with the humerus vertical creating a 90° angle at the elbow joint. Elbow flexor force (N) was measured with an isometric force transducer (TSD121C, 0–100kg range, Biopac Systems Inc., Goleta, CA) placed in series between the orthosis and either the anchor point of the tether or the suspended weight. The force transducer signal was differentially scaled (i.e. calibrated) to measure the MVC (Range 125.7–491.5N) and the 15% MVC target force (Range 13.3–76.2N) to enhance signal resolution at the submaximal force level. The force signal was sampled at 2.5 kHz and smoothed at 200 samples/sec (MP 150, BioPac Systems, Inc. Goletta, CA) then displayed on the computer screen to provide visual feedback to the subject. Elbow joint position (degrees) in the sagittal plane (flexion/extension) was measured with an electrogoniometer (TSD130B Twin-Axis Goniometer 150, (−)90°− (+)90° range, BioPac Systems, Goleta, CA) secured to the skin over the lateral side of the humerus and forearm using double-sided surgical tape. The position signal was sampled at 2.5kHz and displayed as visual feedback.

### Strength Testing of the Elbow Flexors

Elbow flexor strength was defined as the maximal MVC value for the elbow flexors and was assessed at the start of each session and again just after task failure. To establish baseline MVC, subjects performed a minimum of three maximum isometric contractions by pulling against the tethered force transducer. Subjects were instructed to gradually increase their elbow flexion force to maximum over 3-secs and then to hold that maximum force for 3-sec before relaxing. Standard verbal encouragement was provided [1] throughout the contraction and subjects were given visual feedback of their force output on the computer monitor. There was a 1–2 minute rest between each contraction. Subjects performed additional contractions if the MVC trials were not within 5% of each other or if subjects produced more force with each successive trial. One final MVC was performed ∼5-secs after fatigue task failure (5-secs was required to reconnect the tether to the force transducer after the position-matching task). Baseline MVC for each session was defined as the greatest force output from that session and was used to compare to the final MVC performed after task failure. The highest force output during test session 1 was used as the reference level for the 15% MVC target force that was used in both test sessions. Baseline MVC did not differ between test sessions for the entire subject set (n = 10 Test session 1 MVC: 276.39±101.67 N, Test session 1 MVC: 272.0±102.85 N; *p* = 0.40) or for women (n = 5 Test session 1 MVC: 187.05±33.0 N, Test session 2 MVC: 183.90±36.07 N; *p* = 0.56) or for men (n = 5 Test session 1 MVC: 365.72±47.09 N, Test session 2 MVC: 360.39±54.44 N; *p* = 0.42).

### Force-Matching and Position-Matching Fatigue Tasks

During the fatigue-tasks, subjects were asked to sustain a submaximal fatiguing contraction with the elbow flexors equivalent to 15% of their MVC until task failure under two different conditions. Subjects focused their attention on either maintaining a consistent 15% MVC force output during the force-matching task or preventing their elbow joint angle from moving away from 90°-flexion during the position-matching task. Task-specific visual feedback of force output or joint position was displayed as a horizontal line that spanned the width of the computer screen. To optimize real-time performance feedback, a 100-ms time window was used to display the signal permitting the horizontal line to fluctuate above and below the target line with increases and decreases in force output or joint angle. For both fatigue-tasks, subjects were instructed, and reminded throughout task performance, to keep the signal line representing their force output or the elbow joint position as close to the target line for as long as possible. Limb position, including elbow contact on the padded rest, and postural alignment were monitored by close visual inspection and corrective verbal feedback regarding compensations was provided by the same investigator (PSW) for all test sessions. Task failure during the force-matching fatigue-task occurred when subjects could no longer sustain their force output within ±5% of the target force (i.e. 15% MVC±1.50% MVC) or when subjects could not perform the task without compensations for ≥5 seconds. For the position-matching task, task failure occurred when subjects could not keep the elbow joint flexed within ±10° of the 90° target position (i.e. between 80° and 100°) or if, as with the force-matching task, the subjects could not correct their compensations for ≥5 seconds. Typical compensations included forearm supination, shoulder extension, shoulder adduction with external rotation or shoulder abduction with internal rotation. For both tasks, the visual feedback failure criteria spanned a 10-cm bandwidth around the target line on the computer screen, such that the resolution for the visual gain during the force-matching task was 0.5% MVC/cm and 2°/cm during the position-matching task [31], [59], [61].

### Electrical Recordings

Voluntary and evoked EMG signals were recorded from the biceps brachii and the brachioradilais muscles using bipolar surface electrodes (Ag-AgCl, 8-mm diameter, interelectrode distance 25-mm, Trace 1, Nikomed, Huntingdon Valley, PA) located longitudinally over the muscle bellies on shaved, abraded and cleaned skin. The reference electrode was placed on the medial epidcondyle. EMG signals were amplified (1,000x), band-pass filtered (10–500 Hz), and sampled at 2,500 Hz (MP150, BioPac Systems Inc., Goleta, CA).

### Brachial Plexus Stimulation

Electrical stimulation (single pulses, 100-μsec pulse width) to the brachial plexus at Erb’s point in the supraclavicular fossa were delivered using a constant current stimulator (Digitimer D7SAH, Digitimer Ltd., Hertfordshire, UK) to evoke a M*_max_* in the biceps and brachioradialis muscles both before and during the fatiguing contractions. The anodal electrode (Ag-AgCl, 8 mm) was placed on the acromion and the cathodal electrode (Ag-AgCl, 8mm) at the optimal stimulating point in the supraclavicular fossa. The intensity of the electrical stimulus was gradually increased until the evoked M-wave amplitude in the biceps plateaued. A supramaximal stimulus intensity (100–400 mA) equivalent to 120% of the plateau value was used to evoke the M*_max_* response during the 15% MVC baseline and fatiguing contractions. The amplitude of the biceps M_max_ under resting conditions was used as the reference value for the target size of an unconditioned MEP and unconditioned CMEP (i.e.∼50%M_max_, see below) and the mean value did not change significantly between visits (Test session 1∶9.57±2.78 mV, Test session 2∶9.13±3.97 mV; *p* = 0.72).

### Transcranial Magnetic Stimulation

Single and paired monophasic magnetic pulses were delivered over the right motor cortex region for the left upper extremity using a hand-held 70-mm figure-of-8 focal coil connected to a BiStim^2^ stimulator (Jali Medical Inc. Woburn, MA) attached to two Magstim 200^2^ stimulators (The Magstim Co Ltd, Whitland, UK). For cortical paired-pulse protocols one stimulator delivered the conditioning stimulus (CS) and the other the test stimulus (TS). To induce a current field that flowed perpendicular to the central sulcus, from posterior-lateral to anterior-medial direction in the brain, the coil was positioned tangential to the lateral surface of the head and angled 45° from the sagittal plane so that the stimulator handle was pointed in a posterior-lateral direction [62]. With the coil in this location and current flow in this direction, studies evaluating MEP latency, single motor unit behavior, and epidural recordings within the spinal cord have shown that the TMS pulse primarily activates the axons of both excitatory and inhibitory interneurons that then synapse on the corticospinal tract neurons, rather than activating the corticospinal system directly [37], [63]. Therefore, unlike peripheral nerve or cervicomedullary electrical stimulation, which elicits a single synchronous activation of motorneurons, the TMS pulse evokes a descending volley of action potentials in the corticospinal neurons that are temporally summated by the motorneuron pool [64].

The optimal spatial location on the head where the TMS pulse consistently evoked the largest MEP peak-to-peak amplitude in the contralateral biceps muscle at rest (i.e. the motor hotspot) was found by moving the coil in 1-cm steps over the anatomical location of the upper extremity region of the motor cortex while delivering slightly suprathreshold stimuli. Once found, this position was marked with a sticker to ensure consistent coil placement during all TMS protocols for that day. Active motor threshold (AMT) was defined as the minimum stimulator intensity (reported as % of Stimulator Output: % SO) that evoked an MEP with a peak-to-peak amplitude ≥ twice the amplitude of the background interference EMG associated with the 15% MVC force task in at least 50% of the trials [65]. To determine AMT, subjects performed short-duration 15% MVC force-matching contractions (3 to 5-secs) during which a single pulse was delivered. The subject maintained the force output to the target force line after the TMS pulse until instructed to relax. The maximum EMG baseline peak-to-peak amplitude was quantified across the 500 milliseconds prior to the stimulus artifact and averaged across 4 trials. To confirm AMT, the stimulator intensity was varied by 3% of SO and 4 more trials conducted with the process repeating until AMT was determined. AMT values were consistent between test sessions (Test session 1 AMT: 50±9% SO; Test session 2 AMT: 46±8% SO, *p* = 0.17).

The CS intensity used for the first stimulus pulse in the paired-pulse protocols for SICI and ICF was set as 70% of AMT for each session (Test session 1 CS: 35±6% SO, Test session 2 CS: 33±6% SO, *p* = 0.17) [39], [66]. The stimulator intensity was then increased to establish the suprathreshold TS intensity, which was used for single pulse MEP (i.e. unconditioned MEP), the TS (i.e. second stimulus pulse) in SICI and ICF, and for both the CS and TS in the LII paired-pulse protocol. The TS was defined as the stimulus intensity that, when delivered alone during a 15%MVC contraction, was sufficient to evoke an unconditioned MEP amplitude ∼50% of the resting M*_max_* amplitude and with a SP duration greater than 75-msec in the biceps. The value for the TS was consistent between sessions (Test session 1 TS: 75±14% SO, Test session 2 TS: 72±11% SO, *p* = 0.21) and was equivalent to 150±24% of AMT during test session 1 and 157±30% of AMT during test session 2 (*p = *0.22). The interstimulus intervals (ISI) between the CS and TS were 3-msec to assess SICI and 15-msec for ICF. Both LII and paired cortico-cervicomedullary stimulation had a 75-msec ISI. The SP duration was consistent between sessions at baseline (Visit 1∶133±20msec, Visit 2∶129±20 msec, *p* = 0.46) and therefore, sufficiently long enough for the 75-msec ISI used for the LII and paired cortico-cervicomedullary stimulation paired pulse protocols (see below).

### Paired Cortico-Cervicomedullary Stimulation

Direct stimulation of the descending spinal tracts is considered to be the best available method to assess motorneuron excitability because the corticospinal/motorneuron synapse is not modified by Ia presynpatic inhibition, unlike both the H-reflex and the F-wave [46]. Stimulation at the cervicomedullary junction is preferable because there is less of a chance of activating the spinal nerve roots and the bend in the corticospinal tract, as it crosses to the contralateral side of the body in the medullary pyramids, facilitates current activation of the spinal cord tract axons [45]. Recent work comparing the change in MEP and CMEP amplitudes during sustained submaximal fatiguing contractions have found that both the MEP and CMEP amplitudes continue to increase relative to their baseline values and follow a similar pattern of response, but the CMEP amplitude compared to the MEP amplitude (both normalized to M*_max_*) is lower [67]. McNeil et al., using a paired pulse cortico-cervicomedually electrical stimulation protocol where a conditioned CMEP is evoked during the SP after a suprathreshold TMS pulse during an active contraction, found that the conditioned CMEP amplitude progressively declined to below pre-fatigue values during fatiguing maximal contractions and sustained submaximal contractions [22], [23], [47]. They concluded that the motor cortex is not actively inhibited during sustained submaximal contractions but instead that the motorneurons become progressively resistant to stimulation. When evoked during the fatiguing contraction, the CMEP represents the excitability of the spinal motor circuits in the presence of supraspinal drive [22], [23], [47]. If, however, the CMEP is evoked during the SP (analogous to a conditioned MEP in LII), the conditioned CMEP offers a more direct view of the motorneuron responsiveness during fatigue independent of descending inputs [22], [29], [47], [48].

For this experiment, electrical stimulation (single pulse, 100-μsec pulse duration) of the descending spinal tracts at the cervicomedullary junction was delivered using the same constant current stimulator previously described. The electrical current was passed between two self-adhesive surface electrodes (Ag-AgCl, 8 mm) that were affixed to the skin just medial to the mastoid processes on the soft-tissue adjacent to the inferior occiput with the anode on the left side of the spinal column [45]. The stimulus intensity (200–500 mA) was set to evoke, during brief 15% MVC force-matching contractions, an unconditioned CMEP (i.e. CMEP delivered alone) with a peak-to-peak amplitude ∼50% M*_max_*, and equivalent to the MEP amplitude evoked in response to the TS. The mean amplitude for the unconditioned CMEP did not differ between sessions (Test session 1 CMEP: 3.73±1.72 mV; Test session 2 CMEP: 4.57±3.34 mV; *p* = 0.41). As the stimulus intensity increased, the CMEP latency was monitored to be sure it did not “jump” (i.e. sudden 1–2 msec decrease in the latency) indicating that the cervical spinal nerve roots were activated by the stimulus masking the spinal cord tract activation [68]. The paired cortico-cervicomedullary electrical stimulation protocol is analogous to LII, except that the spinal cord stimulation replaces the second TMS pulse [22], [23]. One of the TMS stimulators delivered the first pulse (suprathreshold TS intensity) to the motor cortex to evoke an unconditioned MEP followed by a SP and simultaneously triggered the electrical stimulator to stimulate the cervicomedullary junction after a 75-msec ISI to evoke the CMEP during the SP.

### Data Analysis

For each fatigue-task, the TTF was measured from the recorded torque output signal starting from when the torque signal reached the target force and ending when the failure criteria were met. The amplitude of the voluntary EMG signal was quantified by calculating the root mean squared (RMS) EMG from the biceps and brachioradialis over a 0.5-sec epoch surrounding peak force during the pre-fatigue MVC, prior to the stimulation sequence for the pre-fatigue baseline measures, and prior to the start of each stimulation series during the fatiguing contractions. These data were normalized to the pre-fatigue MVC values for each respective muscle.

The outcome measures for all of the evoked signals were analyzed off-line using the AcqKnowledge software package version 4.2.0 (Biopac Systems Inc. Goleta, CA). Peak-to-peak amplitude, rather than response area, was used to be consistent with recommendations for analyzing TMS protocols [69], [70]. Before further analysis, to control for individual and non-experimental influences on the interpretation of the evoked responses as well as for changes in the muscle fiber excitability associated with fatigue, all evoked responses were normalized to the corresponding M*_max_* amplitude recorded at each time point [27], [71]–[73]. Ratios representing SICI, ICF, and LII were calculated by dividing the corresponding conditioned MEP amplitude by the single-pulse unconditioned MEP amplitude evoked during the same fatigue-stimulus protocol time point. The pre-fatigue values for each dependent variable represent the average of the three baseline measures normalized to the average pre-fatigue M*_max_*; therefore, the pre-fatigue ratio values for SICI, ICF, and LII were calculated from these averages.

**Figure 3:**
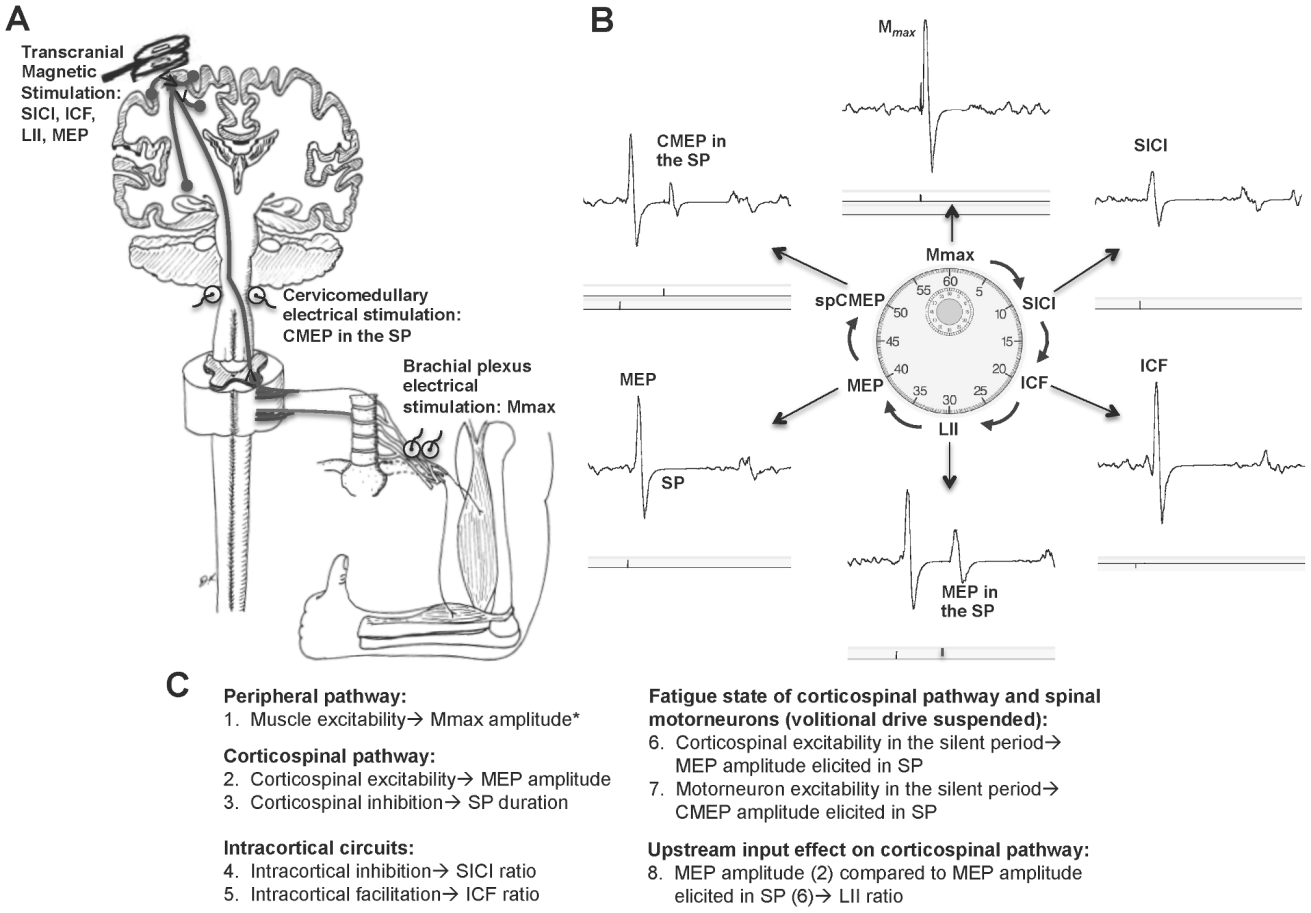
Stimulation sites (A), example evoked potentials (B), and neurophysiologic outcome variables quantified from electromyographic recordings (C). A. Single and paired pulse stimuli were delivered to the motor cortex, the spinal cord at the cervicomedullary junction and the peripheral nerve at the brachial plexus. B. Sample electromyographic recordings of evoked potentials from a single subject elicited by the six stimuli in the protocol sequence. C. Eight neurophysiologic outcome variables were quantified from the evoked potentials. As the SP reflects a temporary cessation of voluntary drive, stimuli delivered during the SP were used to examine the fatigue state of the corticospinal pathway and spinal motorneurons. For intracortical circuits, the amplitude of a conditioned MEP elicited by paired pulse TMS is compared to the amplitude of the single pulse MEP in a ratio. The effect of upstream drive to the motor cortex was examined by comparing the amplitude of the MEP elicited in the SP to the amplitude of the single pulse MEP. This was also quantified as the ratio of long interval inhibition (LII ratio). *All cortical and cervicomedullary evoked potentials were normalized to M*_max._*

In order to assess changes in alpha-motorneuron, corticospinal and intracortical excitability between fatigue-tasks and also relative to each other, all of the evoked outcome variables were calculated as the ratio of the fatigue value divided by the baseline pre-fatigue value, expressed as a percentage [22], [23]. To examine the effect of volitional drive on corticospinal excitability as measured by MEP amplitude during the fatigue-tasks, the 1) normalized MEP evoked in the SP (MEP in SP%pre-fatigue) when volitional drive has been temporarily suspended and the 2) normalized unconditioned MEP (MEP%pre-fatigue) evoked during ongoing volitional output were compared. To examine changes in corticospinal and motorneuron excitability during the SP without volitional drive 3) normalized CMEP evoked in the SP (CMEP in SP%pre-fatigue) was compared with the MEP in SP%pre-fatigue. To compare changes in intracortical inhibition and facilitation the normalized ratios for 4) SICI (SICI%pre-fatigue) and 5) ICF (ICF%pre-fatigue), were examined. To assess changes in the composite measures of corticospinal inhibiton, normalized ratios for 6) LII (LII%pre-fatigue) and 7) SP duration (SP%pre-fatigue) were also compared. **Figure 3** outlines the neurophysiologic outcome variables.

To examine the time-course of changes in the neurophysiologic measures during the fatigue-tasks, the data were compared over time in 20% intervals of the TTF calculated for each individual’s TTF (Pre-fatigue, 20%, 40%, 60%, 80% and 100% of TTF). The absolute times relative to the TTF were found for each individual and the responses measures for the stimuli delivered closest to that time, or the average of two stimuli delivered within an equal time frame on either side of the time point, were used. The last stimulus protocol was delivered within 0.73±0.48 minutes of the TTF and represents the final measure and is thus considered to represent 100% of TTF even if the subject continued to contract for a short period of time after the stimulation protocol was finished.

### Statistical Analysis

SPSS version 20 for Mac (SPSS Inc, Chicago, IL) was used for the statistical analyses. Data are reported as mean±SD in the text with effect size (ES = partial η^2^) and means±SE in the figures. An α of 0.05 was required for statistical significance. A paired *t-*test was used to compare the TTF between the two respective tasks and task-specific baseline values for the neurophysiologic outcomes from each muscle. Two-way repeated measures ANOVA (RM ANOVA) was used to compare changes in MVC with Task (2:Force, Position) and Time (2:pre-fatigue, task failure) as repeated factors. A mixed model ANOVA with Task (2: position, force) as a repeated factor and Gender (2: women, men) was used to compare the TTF between women and men. The effect of target force was analyzed by an ANCOVA (Task, Gender with Target Force covaried). Simple linear regression was then used to examine relationships between TTF and target force.

Analysis of the serial measures of muscle activation (i.e., RMS EMG) during the fatigue-task protocols was conducted using a three-way RM ANOVA with Muscle (Biceps, Brachioradialis), Task and Time (6:Pre-Fatigue, 20,40, 60, 80, Task-Failure) as factors. A significant interaction of Muscle × Time was found for the brachioradialis; thus, the analyses of the eight neurophysiologic outcome variables were conducted separately for the biceps and the brachioradialis. A two-way RM ANOVA with Task and Time (6:Pre-Fatigue, 20,40, 60, 80, Task-Failure) as factors was used to explore for task-specific differences in RPE as well as the eight neurophysiologic outcome variables normalized to pre-fatigue baseline. An interaction for Task × Time was used to examine for differences in rate of change for the outcomes. To compare pairs of outcome variables a three-way RM ANOVA with Task, Time, and Stimulus was used (e.g. Stimulus 2 levels: MEP in SP%pre-fatigue and CMEP in SP%prefatigue). When a significant main effect and/or interaction terms were observed a follow-up post-hoc Sidak test was used to control for alpha inflation.

Sample size for the present study was based on previous data comparing the time to task failure for the force and position matching tasks, and was powered (power = 0.80) to detect significant differences in the time to task failure between the tasks at a P<0.05 [17]. However, with relatively small sample sizes, as in the present study, rather large differences may not reach statistical significance and result in Type II error. Therefore, effect sizes (here, referring to partial eta^2^, which represents the proportion of total variation attributable to the factor, partialling out other factors from the total non-error variation) are reported as an additional statistical parameter to aid in interpretation of the findings.

## Results

### Performance Outcomes

#### Time to task failure and decline in MVC (Figures 4 and 5)

The mean TTF for the position-matching task was 1.5 times longer than the mean TTF for the force-matching task (26.9±15.11 min vs. 17.5±7.9 min, *p*<0.01, ES = 0.60). The decline in MVC following the fatigue tasks did not differ between the two task conditions (Position-matching MVC %-Decline: 30.09±18%, Force-Matching MVC %-Decline: 30.07±15%; MVC Task × Time: *p* = 0.59). Individual values for TTF for the respective tasks are illustrated in Figure 5A. Interestingly, women exhibited a greater magnitude of difference in TTF (TTFDiff_P-F_) between the two fatigue-tasks (Task × Gender: *p<*0.01); however, when the absolute 15% MVC target-force was statistically covaried, the effect of Gender on TTFDiff_P-F_ was no longer significant (Task × Gender: *p* = 0.81; it should be noted that Task Main effect persisted in the covariate analysis, *p<*0.01). There was an inverse relationship between the absolute value for the 15% MVC Target force and the TTFDiff_P-F_ (*r* = –0.85, *p*<0.01). The absolute value for the target force predicted a significant amount of the between-subject variability in TFF for the position-matching task (*r^2^ = *0.81, *p<*0.01) and the force-matching task (*r^2^* = 0.72, p<0.01), as well as for the TTFDiff_P-F_ (*r^2^* = 0.73, p<0.01). Thus, stronger subjects with a higher absolute target force were more likely to have similar TTF between the two tasks, whereas weaker subjects with a lower absolute target force had a greater difference in TTF between tasks with a significantly longer TTF for the position-matching task.

**Figure 4:**
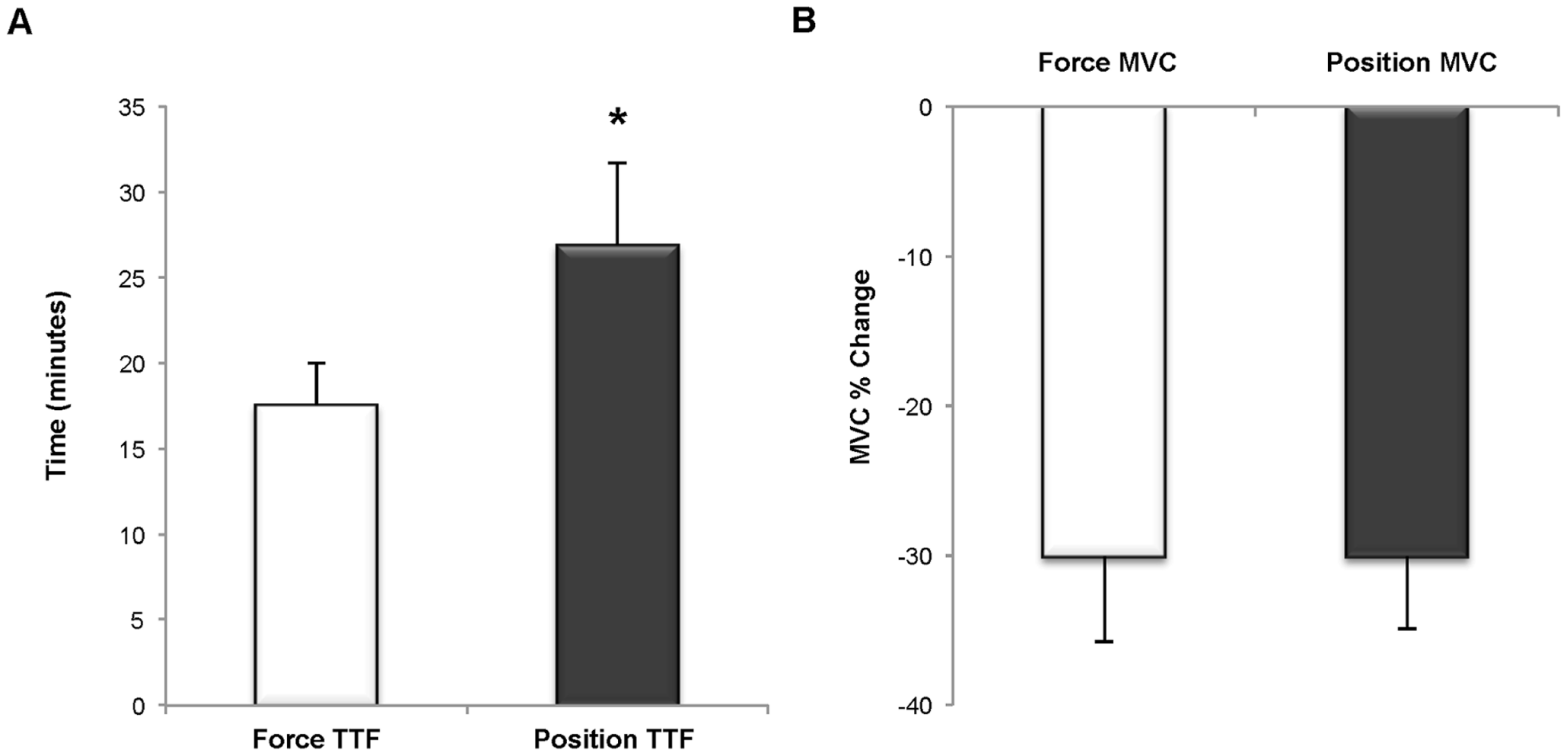
Time to task failure (A) and percent change in elbow flexor maximum voluntary contraction force (B). A. The time to task failure (TTF) for the position-matching task was longer than the TTF for the force-matching task (*p*<0.01, ES = .60). B. There was no difference between the two fatigue-tasks in the amount of muscle fatigue as measured by the percent decline in maximum voluntary contraction (MVC) force at task-failure (*p* = 0.59).

**Figure 5:**
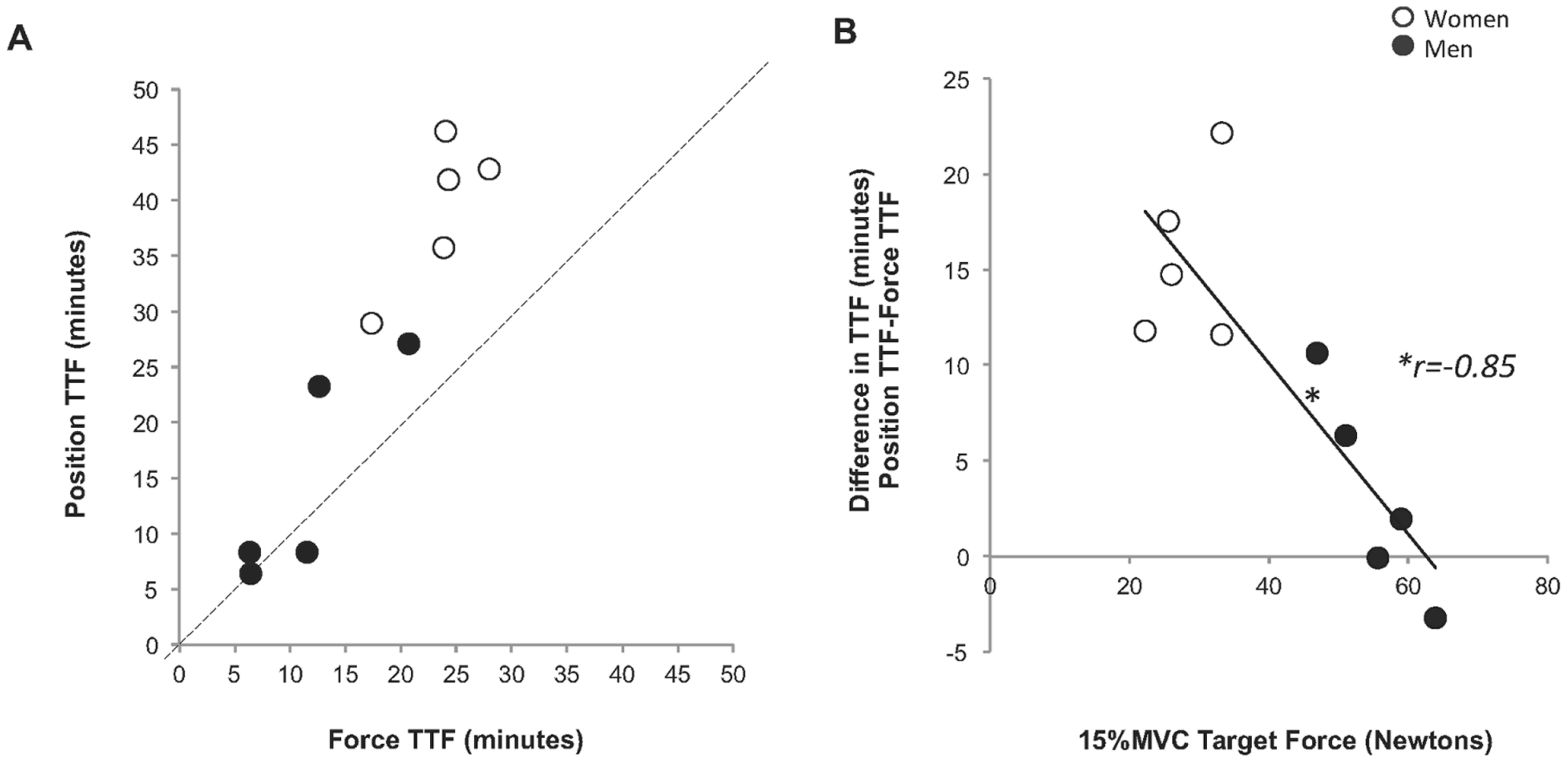
Individual time to task failure (A) and time to task failure difference with target force (B). (*Open circles*) Women (*Filled circles*) Men A. When compared to the force-matching task, 80% of the subjects had a greater time to task failure (TTF) during the position-matching task. The dashed line represents an equivalent TTF for both fatigue-tasks. B. There was inverse relationship between the magnitude of the difference in TTF (position-matching TTF – force-matching TTF) of the two fatigue-tasks and the absolute value of the target force (*r = –0.85, *p<0.01*).

#### Rating of perceived exertion

RPE increased throughout both fatigue tasks from 0 to equivalent mean values of 9.5±0.26 during the force-matching task and 9.7±0.21 for the position-matching task (Time *p*<0.01, ES = 0.96). The RPE values between pre-fatigue and 80% of TTF were significantly greater than the previous interval (post hoc pairwise comparison *p*<0.01) and then plateaued between 80% and 100% TTF (*p = *0.72).

### Neurophysiologic Outcomes

Pre-fatigue baseline measures of muscle activation and the eight neurophysiologic outcome variables for the biceps and brachioradialis are presented in **Table 1**. Prior to the fatigue tasks, the SP duration was significantly longer in the position-matching task than the force-matching task for both muscles (*p*<0.001). There were no significant differences in the pre-fatigue measures, in either the biceps or the brachioradialis, for muscle activation (*p*>0.10), the amplitudes for M*_max_* (*p*>0.10), unconditioned MEP (*p*>0.05), conditioned MEP elicited in the silent period (MEP in SP: *p>*0.10), and conditioned CMEP elicited in the SP (CMEP in SP: *p*>0.10) nor were there significant baseline differences in the calculated ratios for SICI (*p*>0.10), ICF (*p*>0.05), and LII (*p*>0.10).

**Table 1:** Pre-fatigue baseline values for the neurophysiologic outcome variables.

BICEPS				BRACHIORADIALIS			
*Force*	*Position*	*p*	ES	*Force*	*Position*	*p*	ES
**RMS EMG %pre-fatigue MVC**							
13.07±6.43	11.27±6.83	0.39	0.08	13.24±5.01	10.22±3.36	0.06	0.33
**M** ***_max_*** ** mV**							
6.69±3.60	9.74±4.78	0.11	0.26	7.50±.4.65	8.20±3.73	0.67	0.02
**MEP %M** ***_max_***							
69.49±28.65	51.93±15.83	0.08	0.31	49.63±25.99	44.98±24.68	0.27	0.13
**SP duration msec**							
127.97±19.69*	146.47±22.19*	**0.00**	0.74	127.97±26.48*	150.20±27.48*	**0.00**	**0.67**
**MEP in SP %M** ***_max_***							
44.58±21.52	30.03±18.88	0.10	0.28	31.82±17.95	25.00±19.38	0.16	0.21
**CMEP in SP %M** ***_max_***							
46.84±29.98	33.92±26.99	0.19	0.18	36.43±21.52	26.29±23.06	0.19	0.19
**SICI ratio**							
0.75±0.27	0.69±0.27	0.55	0.04	0.87±0.29	0.78±0.32	0.16	0.20
**ICF ratio**							
1.04±0.18	1.07±0.17	0.61	0.03	1.30±0.39	1.01±0.11	0.07	0.33
**LII ratio**							
0.73±0.48	0.60±0.41	0.14	0.23	0.77±0.46	0.55±0.34	0.12	0.25

Data are mean values (±SD) of the three pre-fatigue baseline measures taken during short duration 3–5sec 15%MVC task-specific contractions. Asterisks (*) signify a task-specific difference between baseline values (*p* values <0.05 are indicated by bold text). *ES: Effect size partial eta^2^; ICF: intracortical facilitation; LII: long interval inhibition; MEP: motor evoked potential; Mmax: maximum compound muscle action potential; MVC: maximum voluntary contraction; RMS EMG: root mean squared electromyographic amplitude; SICI: short interval intracortical inhibition; SP: silent period; spCMEP: cervicomedullary evoked potential elicited in the silent period.*

#### Muscle activation (Figure 6)

There were no differences in the amounts of muscle activation throughout the performance of the two fatigue tasks to task failure (Task × Muscle × Time: *p* = 0.21, ES = 0.16); however, the rate of muscle activation was greater for the brachioradialis (Muscle × Time: p = 0.03 ES = 0.31). Although the amount of muscle activation in the brachioradialis was greater during the force matching task, the difference was not statistically significant (Muscle × Task: *p = *0.19, ES = .0.18; Muscle: *p* = 0.08, ES = 0.31). At task failure the Biceps EMG had increased to 25.95±10.88%MVC and 28.12±17.21%MVC and the Brachioradialis EMG increased to 39.30±18.31%MVC and 27.59±13.23%MVC for the force-matching and position-matching tasks respectively (Time: p = 0.00 ES = 0.78; Sidak post hoc test p<0.05 between all TTF intervals except 80%TTF vs.100%TTF).

**Figure 6:**
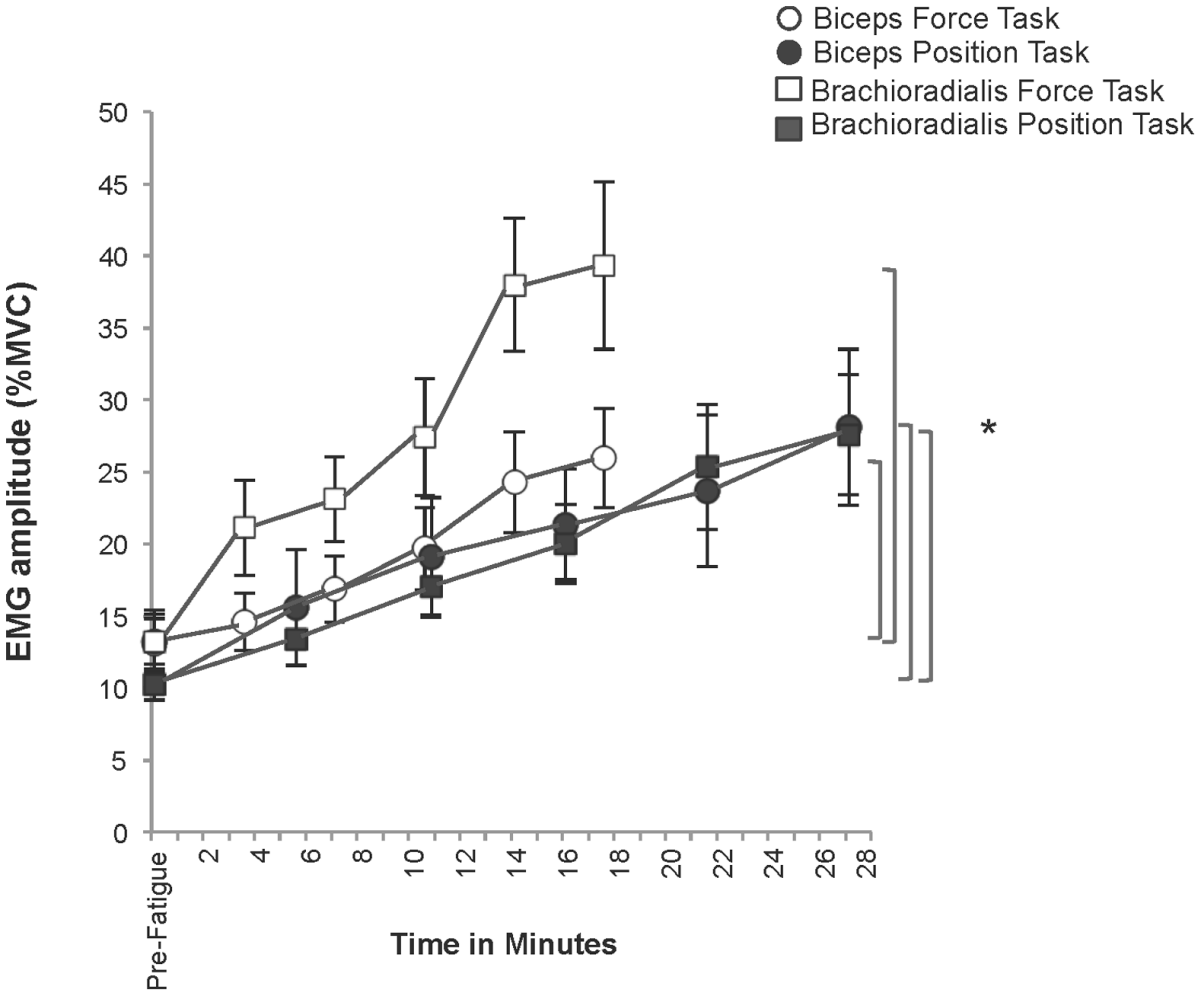
Muscle activation of the biceps and brachioradialis during the force-matching and position-matching tasks. The EMG amplitude (% of pre-fatigue MVC) increased in both muscles throughout both fatigue tasks (*p = 0.00) but did not differ between tasks or muscles. The rate of muscle activation was greater for the brachioradialis during fatigue task performance (*p* = 0.03). Each data point represents the mean EMG (% of pre-fatigue MVC) at 20% intervals of the TTF relative to each individual’s TTF during each task. **The respective TTF interval was significantly greater than all previous intervals except 80% vs. 100%TTF.

#### M_max_

During the fatigue-tasks, the M*_max_* amplitude did not change throughout either fatigue-task (Biceps Time: *p* = 0.38 ES = 0.10; Brachioradialis Time: *p* = 0.74 ES = 0.03) nor differ significantly between fatigue-tasks (Task: Biceps *p = *0.68 ES = 0.02; Brachioradialis *p* = 0.07 ES = 0.33).

#### MEP amplitude (Figure 7A)

The amplitude of the unconditioned MEP gradually increased in the biceps to 157.60±85.33%pre-fatigue baseline and to 147.60±79.57%pre-fatigue baseline (Time: *p* = 0.03, ES = 0.33) and in the brachioradialis to 226.13±153.49%pre-fatigue baseline and to 223.80±125.95%pre-fatigue baseline (Time: *p* = 0.01, ES 0.41) by task failure for the force-matching and position-matching tasks respectively. There were no significant post hoc pair-wise comparisons for Time (*p*>0.05). No task-specific differences were found for the amount of increase in MEP amplitude (Task: Biceps *p* = .91 ES = 0; Brachioradialis *p* = 0.38 ES = 0.09) or for the rate of change in MEP amplitude (Task × Time: Biceps *p* = 0.82 ES = 0.03; Brachioradialis *p* = 0.56 ES = 0.07).

**Figure 7:**
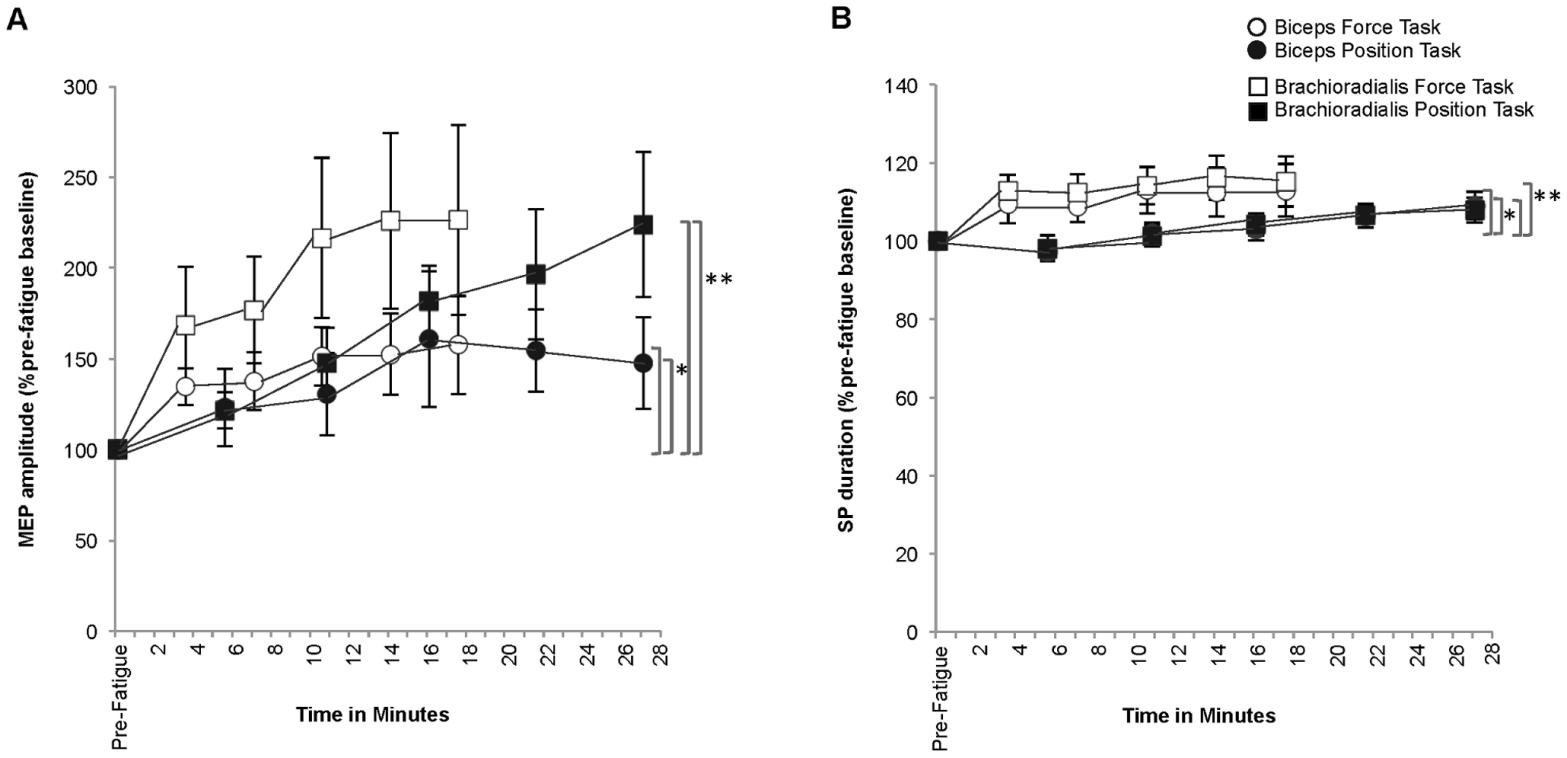
Motor evoked potential amplitude (A) and silent period duration during the force-matching and position-matching tasks (B). A. The motor evoked potential (MEP) amplitude (%pre-fatigue baseline) increased during both fatigue-tasks in the biceps (*p = 0.03) and the brachioradialis (**p = 0.01). B. Silent period (SP) duration (%pre-fatigue baseline) increased throughout both fatigue tasks in the biceps (*p<0.01) and the brachioradialis (**p<0.01).

#### Silent period duration (Figure 7B)

At task failure, the SP duration had increased in the biceps to 112.97±6.68%pre-fatigue baseline and to 109.11±3.53%pre-fatigue baseline (Time: *p*<0.01, ES = 0.37) and in the brachioradialis to 115.25±6.36%pre-fatigue baseline and to 107.97±3.17%pre-fatigue baseline (Time: *p* = 0.02, ES = 0.36) for the force-matching and position-matching tasks respectively. There were no significant post hoc pair-wise comparisons for Time (*p*>0.05). There were no task-specific differences for the amount of change (Task: Biceps *p* = 0.24 ES = 0.15; Brachioradialis *p* = 0.07 ES = 0.34) or for rate of change in SP duration (Task × Time: Biceps *p = *0.33 ES = 0.12; Brachioradialis *p = *0.13 ES = 0.18).

#### MEP elicited during the silent period (Figure 8A)

During both fatiguing contractions, the amplitude of the MEP in SP gradually increased in the biceps to 197.22±41.60%pre-fatigue baseline and to 164.74±35.57%pre-fatigue baseline (Time: *p* = 0.01, ES = 0.30) and in the brachioradialis to 175.77±30.39%pre-fatigue baseline and to 193.41±36.66%pre-fatigue baseline (Time: *p* = 0.01, ES 0.38) for the force-matching and position-matching tasks respectively. There were no significant post hoc pair-wise comparisons for Time (*p*>0.05). No task-specific differences were found for either the amount of change (Task: Biceps *p* = .83 ES = 0.01; Brachioradialis *p* = 0.86 ES = 0) or the rate of change in MEP in SP amplitude (Task × Time: Biceps *p = *0.53 ES = 0.08; Brachioradialis *p = *0.78 ES = 0.03).

**Figure 8:**
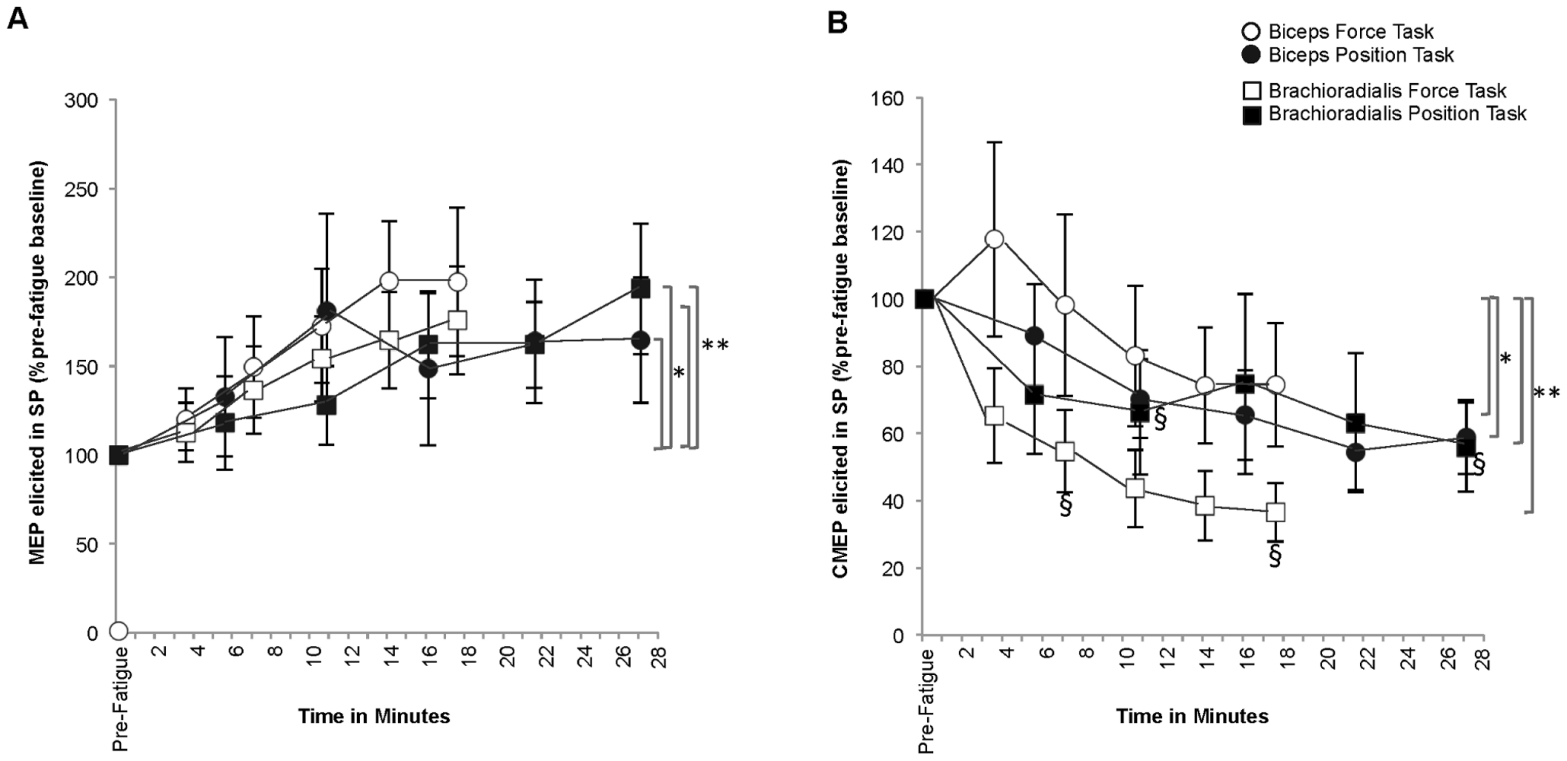
Cortically-evoked response elicited in the silent period (A) and cervicomedullary-evoked response elicited in the silent period (B). A. The amplitude (% of pre-fatigue baseline) of the motor evoked potential elicited in the silent period (MEP elicited in SP) increased progressively to task failure during both fatigue-tasks in the biceps (*p = 0.01) and the brachioradialis (**p = 0.01). B. The amplitude (%pre-fatigue baseline) of the cervicomedullary-evoked potential elicited in the SP (CMEP elicited in SP) progressively decreased throughout the fatiguing contractions to task failure in the biceps (*p = 0.02) and the brachioradialis (**p<0.001).

#### CMEP elicited in the silent period (Figure 8B)

The amplitude of the CMEP in SP gradually decreased in the biceps to 74.41±18.40%pre-fatigue baseline and to 58.93±11.01%pre-fatigue baseline (Time: *p* = 0.02, ES = 0.35) and in the brachioradialis to 35.42±8.72%pre-fatigue baseline and to 55.89±13.19%pre-fatigue baseline (Time: *p*<0.00, ES = 0.52) for the force-matching and position-matching tasks respectively at task failure. Significant pairwise comparisons for Time were found in the brachioradialis between pre-fatigue and the 40%TTF and 100%TTF intervals. There were no significant post hoc pair-wise comparisons for Time in the biceps (*p*>0.05). There were no task-specific differences in the overall amount of change (Task: Biceps *p* = 0.36 ES = 0.09; Brachioradialis *p* = 0.40 ES = 0.08) or in the rate of change for CMEP in SP amplitude (Task × Time: Biceps *p* = 0.61 ES = 0.06; Brachioradialis *p* = 0.52 ES = 0.07).

#### Short-Interval intracortical inhibition ratio (Figure 9A)

In both fatiguing contractions, the SICI ratio increased to 146.63±22.81%pre-fatigue baseline in the force-matching task and to 191.72±41.40%pre-fatigue baseline for the position-matching task (Time: *p* = 0.02, ES = 0.35) indicating decreasing intracortical inhibition in the biceps. There were no significant post hoc pair-wise comparisons for Time (*p*>0.05). The changes in the SICI ratio for the brachioradialis, while greater than baseline, were not statistically significant (force-matching: 113.61±16.01%pre-fatigue baseline, position-matching: 120.23±13.02%pre-fatigue baseline; Time: *p* = 0.12 ES = 0.17). There were no task-specific differences in the amount of change (Task: Biceps *p* = .52 ES = 0.05; Brachioradialis *p* = 0.26 ES = 0.14) or the rate of change in SICI ratio (Task × Time: Biceps *p* = 0.58 ES = 0.06; Brachioradialis *p* = 0.52 ES = 0.07).

**Figure 9:**
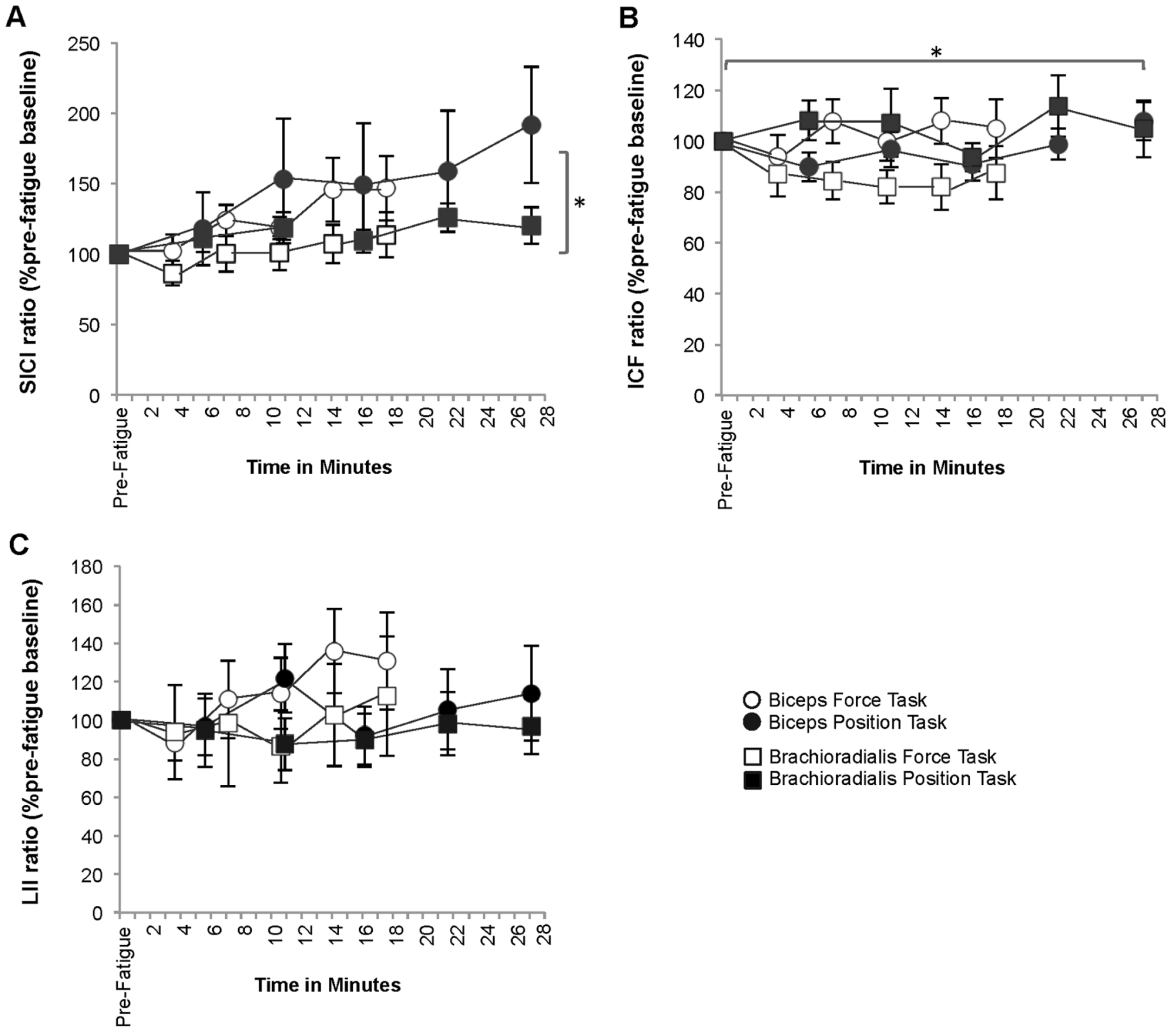
Short interval intracortical inhibition ratio (A), intracortical facilitation ratio (B) and long-interval inhibition ratio (C). A. The value for the ratio (%pre-fatigue baseline) of short interval intracortical inhibition (SICI ratio) progressively increased throughout the fatigue-tasks in the biceps (*p = 0.03), which is consistent with *decreasing* intracortical inhibition and did not change in the brachioradialis (*p*>0.10). B. The value for the intracortical facilitation (ICF) ratio (%pre-fatigue baseline) did not change during fatigue-task performance in either muscle (*p*>0.10) and, in the brachioradialis, remained greater than baseline during the position-matching task and less than baseline in the force-matching task (*p = 0.20). C. The value for long interval inhibition (LII) ratio (%pre-fatigue baseline) did not differ between the fatigue-tasks nor change during fatigue-task performance (*p*>0.50).

#### Intracortical facilitation ratio (Figure 9B)

The ICF ratio did not significantly change in either muscle during either fatiguing contraction (Time: Biceps *p* = 0.10 ES = 0.18; Brachioradialis *p* = 0.69 ES = 0.04). There was a task-specific difference in the overall value for the ICF ratio in the brachioradialis with the mean value throughout the force-matching task (87.11±16.44%pre-fatigue baseline) significantly lower than during the position-matching task (104.69±12.65%pre-fatigue baseline; Task: *p* = 0.02, ES = 0.47), but not for the biceps (Task: *p* = 0.40, ES = 0.08). There was no difference in the rate of change in ICF ratio (Task × Time: Biceps *p* = 0.72 ES = 0.06; Brachioradialis *p* = 0.21 ES = 0.15).

#### Long interval inhibition ratio (Figure 9C)

There were no changes in the LII ratio throughout the fatiguing contractions (Time: Biceps *p* = 0.41 ES = 0.09, Brachioradialis *p* = 0.91 ES = 0.03) nor were there any task-specific differences in the overall value for the LII ratio (Task: Biceps *p* = 0.55 ES = 0.04, Brachioradialis *p* = 0.82 ES = 0.01) in either muscle.

#### MEP vs. MEP elicited in the silent period (Figure 10A)

Measures of corticospinal excitability with (MEP) and without volitional drive (MEP in SP) progressively increased in both muscles throughout both fatigue tasks to task failure (Time: Biceps MEP: 152.62±20.71%pre-fatigue baseline, MEP in SP:181.03±29.52%pre-fatigue baseline *p* = 0.00, ES 0.41; Brachioradialis MEP: 224.89±41.98%pre-fatigue baseline, MEP in SP 184.14±28.05%pre-fatigue baseline *p* = 0.01, ES = 0.46). There were no significant differences between tasks and stimuli in either muscle (Stimulus × Task × Time: Biceps *p* = 0.23 ES = 0.14; Brachioradialis *p* = 0.67 ES = 0.04).

**Figure 10:**
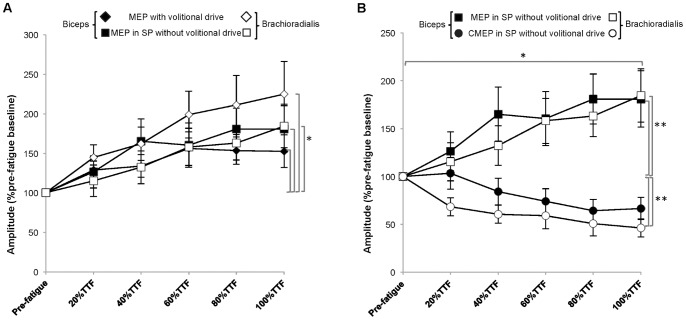
Comparisons of corticospinal excitability with and without volitional drive (A) and corticospinal and spinal excitability without volitional drive (B). A. The amount of corticospinal excitability (%pre-fatigue baseline) both with volitional drive (MEP) and without volitional drive (MEP in SP) increased throughout fatigue task performance in both muscles (*p<0.01). There were no differences found between tasks; therefore, values were pooled across tasks for clarity. B. The amount of corticospinal excitability (%pre-fatigue baseline) without volitional drive (MEP in SP) progressively increased and was greater throughout task performance in both muscles (*p<0.001) relative to the amount of spinal excitability (%pre-fatigue baseline) without volitional drive (CMEP in SP) which progressively decreased throughout fatigue-task performance in both muscles (**p = 0.00). There were no differences found for task therefore, values were pooled across tasks for clarity.

#### MEP elicited in the silent period vs. CMEP elicited in the silent period (Figure 10B)

Measures of the fatigue state of corticospinal excitability (MEP in SP), were significantly greater throughout fatigue task performance relative to the fatigue state measures of spinal excitability (CMEP in SP) (Stimulus × Time: Biceps *p* = 0.00, ES 0.42; Brachioradialis *p* = 0.00, ES = 0.61), but there were no differences between tasks (Stimulus × Task × Time: Biceps *p* = 0.34 ES = 0.12, Brachioradialis *p* = 0.87 ES = 0.04). The mean MEP elicited in SP was significantly greater than the mean CMEP elicited in the SP during fatigue task performance (Stimulus: Biceps MEP in SP152.32±19.32%pre-fatigue baseline, CMEP in SP 82.12±10.11%pre-fatigue baseline, *p* = 0.17, ES = 0.49; Brachioradialis MEP in SP 142.30±17.05%pre-fatigue baseline, CMEP in SP 64.05±8.42%pre-fatigue baseline, *p* = 0.00, ES = 0.77).

#### SICI ratio vs. ICF ratio (Figure 11A)

In the biceps, the mean value for SICI increased to 169.20±25.5% of pre-fatigue baseline during the fatigue-tasks while the ICF ratio did not change (106.4±7.72%pre-fatigue baseline) indicating a reduction in short interval intracortical inhibition during the fatigue tasks with sustained intracortical facilitation (Stimulus × Time: *p* = 0.01, ES = 0.46). There were no significant differences found for the brachioradialis (*p*>0.05).

**Figure 11:**
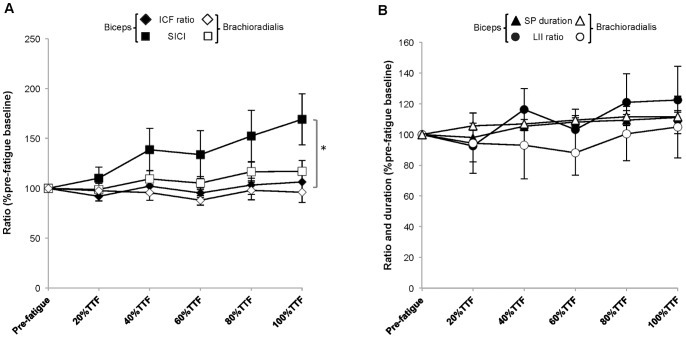
Comparisons between intracortical inhibition and facilitation (A) and long interval inhibition with silent period duration (B). A. In the biceps, the amount of short interval intracortical inhibition (SICI ratio %pre-fatigue baseline) increased during fatigue task performance indicating a *reduction* in inhibition while the amount of intracortical facilitation (ICF ratio %pre-fatigue baseline) remained unchanged (*p = 0.01); however, there were no differences found in the brachioradialis for either variable (*p*>0.05). Values were pooled across tasks for clarity as there were no differences found by task. B. Comparison of the ratio for long interval inhibition (LII as %pre-fatigue baseline) and the duration of the silent period (SP duration as %pre-fatigue baseline) did not demonstrate significance when compared by task, time and stimulation protocol suggesting no change in composite corticospinal inhibition. Values were pooled across tasks for clarity.

#### LII ratio vs. SP duration (Figure 11B and Figure 12)

Initial analyses comparing the LII ratio with the SP duration did not reveal any significant effects (*p*>0,05); therefore, the LII ratio was further analyzed individually. During the fatiguing contractions, the mean value for the normalized LII ratio in the biceps was 113.3±10.9%pre-fatigue baseline and 105.0±11.4% and in the brachioradialis 99.0±20.8%pre-fatigue baseline and 94.5±10.7%pre-fatique baseline during the force-matching task and position-matching tasks respectively which suggests no increase in corticospinal inhibition during the fatigue tasks (Task × Time: Biceps *p* = 0.62 ES = 0.06, Brachioradialis *p* = 0.78 ES = 0.34).

**Figure 12:**
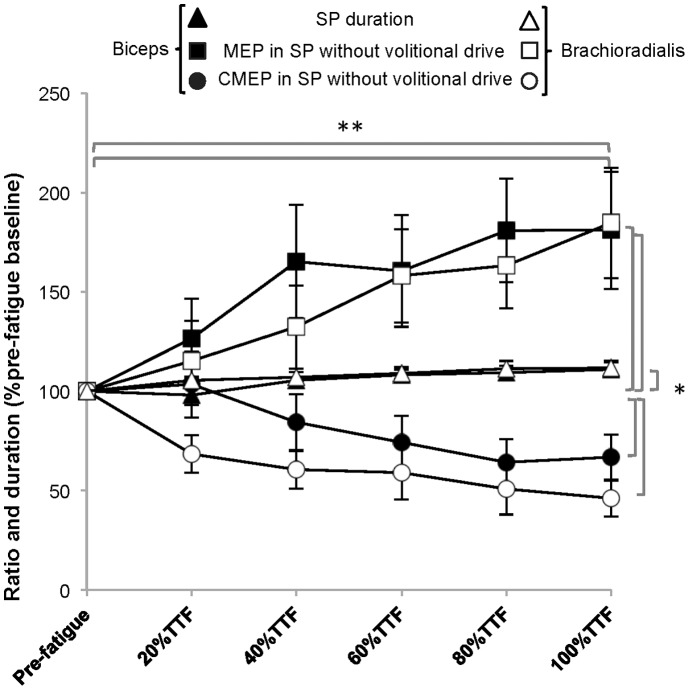
Comparisons between silent period duration with corticospinal and spinal excitability measured during the silent period. The duration of the silent period (SP duration %pre-fatigue baseline) progressively increased during fatigue task performance to task failure as the amount of corticospinal excitability (%pre-fatigue baseline) measured during the silent period (MEP in SP without volitional drive) progressively increased and the amount of spinal excitability (%pre-fatigue baseline) measured in the silent period (CMEP in SP without volitional drive) progressively decreased in both muscles (*p = 0.00). The value for the MEP elicited in the SP as well as the SP duration remained above baseline and were significantly greater than the mean value for the CMEP elicited in the SP which was below baseline for both muscles (**p<0.05).

To explore the relationship between SP duration and measures of corticospinal and motorneuron excitability in the SP, a final 3-way RM-ANOVA (Task (2), Time (6), Stimulus (3)) comparing the normalized values for SP duration, CMEP evoked during the SP, and MEP evoked during the SP was completed (**Figure 12**) in the biceps and the brachioradialis. This analysis revealed that during the fatiguing contractions, in the biceps as the SP duration increased to 111.0±3.4%pre-fatigue, the MEP in SP increased to 181.0±29.5%pre-fatigue while the CMEP in SP amplitude decreased to 66.7±11.6% of pre-fatigue values (Stimulus × Time: *p* = 0.00, ES 0.36). Similar results were found in the brachioradialis (SP duration: 111.6%±3.8%pre-fatigue baseline, MEP in SP: 184.6±27.7%pre-fatigue baseline, CMEP in SP: 46.2±9.3%pre-fatigue baseline; Stimulus × Time: *p* = 0.00, ES 0.50). Significant pairwise comparisons for Stimulus were found between the CMEP elicited in the SP with the MEP elicited in the SP and the SP duration in both muscles greater overall (p<0.05).

## Discussion

The purpose of this study was to compare the task specific differences in the adjustments in cortical and spinal excitability that developed during the performance of the force-matching and position-matching tasks with the elbow flexors in order to further delineate the contribution of supraspinal mechanisms to task failure during sustained submaximal contractions. There were five main findings from this experiment. First, contrary to expectations, the duration of the position-matching task with the elbow flexors was 45% longer than the force-matching task when performed under identical mechanical demands without proximal stabilization or restraint of the forearm. Second, there were no significant task-specific differences found for the total amount of change and the rate of change for the eight neurophysiologic outcome variables in the biceps muscle. Third, the amount of corticospinal excitability (i.e., MEP and MEP elicited in the SP) increased throughout fatigue task performance while the amount of spinal excitability (i.e., CMEP elicited in the SP) decreased. Fourth, the amount of intracortical inhibition within the motor cortex decreased or was sustained (i.e., SICI ratio) during fatigue task performance. Both ICF within the motor cortex (i.e., ICF ratio) and upstream excitation of the motor cortex (i.e. MEP vs. MEP elicited in the SP, LII ratio) remained constant. Lastly, the increase in the duration of the SP followed the progressive decrease in spinal excitability throughout fatigue-task performance but deviated from the progressive increase in corticospinal excitability. Below, these key results will be discussed further.

### Task-specific Differences in Performance

Consistent with the paradigm identified in prior studies with the elbow flexors comparing the task duration of two sustained submaximal contractions that differ by load compliance, both tasks resulted in the same amount of muscle fatigue, amount of muscle activation, and change in RPE despite significant differences in contraction times [15], [59]. Contrary to our initial hypothesis, the duration of the position-matching task was found to be nearly 42% longer than the duration of the force-matching task. Therefore, the shorter TTF for the force-matching task observed here is in disagreement with the majority of the current literature comparing the task duration between the force-matching and position-matching tasks [11], [16], [17], [27], [50], [57], [59], [74]–[78].

Prior studies examining sustained submaximal contractions (i.e. ≤30%MVC) of the elbow flexors with the upper extremity positioned next to the body, the elbow flexed to 90° and the forearm in neutral as in this study have consistently found the position-matching task TTF to be on average 40% *shorter* than the force-matching task TTF [16], [17], [27], [57], [59]. The most common results reported for studies comparing the TTF for the two tasks performed by extremity muscles (i.e. ankle dorsiflexors, knee extensors, wrist extensors, first dorsal interossei) [11], [50], [74]–[76], [78] and for the elbow flexors in a different arm posture [57], [77], [79] are for a 21–53% increase in TTF for the force-matching task depending upon contraction intensity and muscle tested. However, while most common, the paradigm is far from ubiquitous and has several caveats. The magnitude of difference in TTF for the force-matching task over the position-matching task has been found to depend upon: 1) the intensity of the submaximal contraction [15], [16], [75]; 2) the posture of the limb and the body [57], [77]; and 3) the amount of proximal stabilization and limb support provided during task performance [11], [76], [80]. The effect of these conditions is to reduce and even eliminate the difference in TTF between the two tasks due to the total amount of muscle activation required by not only the primary movers but also synergists, accessory muscles and postural stabilizers [16], [57], [74]. Thus, while this is not the first study to find results about task duration that differ from the most common result, this is the first extremity muscle study to report the direct opposite result: the TTF for the position-matching task with the elbow flexors was 42% longer than the force-matching task. Recently, one study evaluating the trunk extensors has also reported similar results [79]. Two aspects of the experimental setup used in this study may have contributed to the reversal in task duration result: 1) subject stabilization and 2) sensitivity of the visual feedback.

Prior to discussing the factors that could explain the unexpected TTF, to ensure the validity of the experimental strategy and therefore its usefulness for addressing the stated hypotheses about neural mechanisms of task failure in sustained submaximal contractions, it is worth highlighting the performance results that were consistent with prior force-matching/position-matching studies. First, despite the differences in TTF, the amount of muscle fatigue, as measured by the decline in MVC force at task failure, was similar for the two tasks (i.e. 30±15% and 30±18%) and comparable to the results reported from prior studies of the elbow flexors using a 15% MVC [17], [59]. Second, at task failure, the amount of perceived effort reported by subjects, using the RPE scale, increased to the same value for both tasks. Therefore, consistent with the paradigm, at task failure both tasks ended with the same amount of physical and perceptual fatigue/exertion despite having a 42% difference in task duration [12], [15], [16], [59]. Third, consistent with the intensity/duration characteristics of sustained submaximal contractions (i.e. lower intensity contractions have a longer duration), the absolute target force the individual subjects exerted with the elbow flexors was a significant contributor to the TTF for both the force-matching and the position-matching tasks [18], [55] and eliminated the gender differences found for TTF [81]. Additionally, the magnitude of the absolute target force explained 73% of the variability in the difference in TTF between the two tasks. The stronger subjects who sustained a higher absolute target force were more likely to have similar TTF for both tasks, whereas weaker subjects with a lower absolute target force had a greater difference in TTF between tasks with a significantly longer position-matching TTF. The difference in TTF has been attributed to faster and/or greater recruitment of the motor pool during the shorter task [17], [31]. Therefore, for the stronger subjects with a higher magnitude absolute target force, most likely a greater proportion of the motor pool was recruited at the start of both fatigue tasks in order to produce the force output needed for task performance leaving fewer unrecruited motor units available to be recruited as the contraction duration progressed thus eliminating the difference in TTF.

### Task Specific Differences in Measures of Supraspinal and Spinal Excitability

There were no differences found between the force-matching and the position-matching tasks for either the mean total amount of change or the rate of change for the eight neurophysiologic measures of segmental excitability in the biceps. Additionally, there were no significant task-specific differences in the total amount of change in muscle activation for both muscles. Although the brachioradialis had a greater rate of activation during fatigue task performance, this difference was not task specific. The only significant task-specific difference was found in the brachioradialis muscle for the ICF ratio where the mean value was greater than baseline for the position-matching task and less than baseline throughout the force-matching task; however, this value did not change with fatigue. Thus, it is unlikely that this one difference could explain the difference we found in the TTF between the force-matching and position-matching tasks because the value for ICF did not change as fatigue developed and remained constant to task failure. Instead, this finding may suggest a task-specific difference in intracortical control mechanisms of the brachioradialis necessary for performance but unrelated to fatigue [20], [37]. Together these results suggest that the net amount and rate of change in segmental excitability, as measured by the eight variables, that developed with fatigue did not differ between the two tasks.

The only prior study comparing cortical and spinal excitability measures between the two tasks found equivalent changes in corticospinal excitability, as measured by MEP amplitude, and a task specific difference in spinal excitability, measured with the H-reflex, such that the shorter duration position-matching task had both a greater and faster decline in spinal excitability [27]. The shorter duration of the position-matching task was ascribed to a decline in neural activation of the muscle secondary to a decrease in peripheral sensory afferent drive to the motor pool from the muscle spindle Ia afferent fiber due to the effect that inertial load compliance has on the sensitivity of the stretch reflex–this as opposed to a reduction in descending motor drive or adaptations intrinsic to the motorneuron that modulate firing rates [5], [15], [17], [27], [74]. This disfacilitation is suggested to be mediated by presynaptic inhibition of the Ia afferent fiber by descending inputs thereby permitting more sensory feedback to be delivered to supraspinal centers which can then be used to adjust the drive to the motor pool via long loop reflex control [17], [27]. Evidence to support this hypothesis include the task-specific differences in H-reflex amplitude [27] and decline in Ia presynaptic inhibition (i.e., less inhibition) during the longer duration force-matching task [17]. The purpose of the increased presynaptic inhibition in the position-matching task is suggested to permit greater cortical influence over the motor pool to minimize force fluctuations [15], [82].

Prior studies using the force-matching/position-matching task paradigm to investigate neural mechanisms associated with task failure have suggested that there are fundamental differences in central neural control strategies that are driven by the task demands, specifically the compliance or stiffness of the load [5], [7], [11], [12], [15], [16], [57], [59], [74]. This same rationale of a different neural control strategy may explain the shorter duration found in this study for the force-matching task and, in addition to differences in load compliance, could be driven by two specific elements of the experimental setup used in this study: 1) the absence of stabilization and limb constraint during task performance and 2) the sensitivity of the visual feedback.

It is well understood that less stabilization requires that more muscles are used to perform the task and if more muscles perform the task, then there is an overall greater level of central neural drive [5], [16], [57], [83]. Greater stabilization has been associated with less muscle activation of prime movers and accessory muscles as well as a longer TTF for the force-matching task [16], [57], [74], [77]. It should be noted, that to date, co-activation ratios between the agonist prime movers (e.g., biceps) and antagonists (e.g., triceps) have been found to be the same between the two tasks and therefore do not to contribute to the differences in TTF [27], [67], [74], [75]. In this study, every effort was made to ensure equivalent mechanical demands during task performance. Therefore, external supports (e.g., straps) were not used to restrict motions at the shoulder or the torso in order to equalize the demand placed on synergists, proximal joints, and postural stabilizers between the two fatigue-tasks. Additionally, in both tasks the arm was free to move in all degrees of freedom with the tether used in force-matching task only limiting the amount of elbow flexion. This is different the typical setup used for the force-matching task where the orthosis is clamped to a frame that effectively holds the forearm in the target position and prevents motion [11], [16], [17], [27], [50], [57], [59], [74], [75], [77], [78]. Accordingly, the shorter task duration found in this study for the force-matching task could be related to the overall amount of muscle activity needed within the upper extremity and torso to perform the task.

Two studies using lower limb muscles have compared the TTF across three conditions: the typical free-motion position matching task, a more restrained position-matching task, and the typical setup for the force-matching task [11], [76]. When a greater amount of restraint was provided to the limb during the position-matching task, the difference in TTF between the force-matching and position-matching tasks was reduced by half [11], [76]. This suggests that with more restraint there were less degrees of freedom to control during the position-matching task as well as less compensatory movements to prevent at other joints. One additional “benefit” of restraint and stabilization from external supports during task performance is that it is easier for synergist muscles from both proximal and distal joints to assist in isometric task performance. Indeed, the intention of effective stabilization during isometric task performance is to minimize joint motion that could occur at other joints; however, just because the joint motions are restricted does not mean that these other muscles are not producing force. For example, greater activation was recorded in the rectus femoris muscle during the force-matching task performed by the dorsiflexors. It was suggested that the hip flexion action of the rectus femoris could assist in task performance as both the foot and leg were restrained [74].

In this study, because the subjects were unrestrained, they were closely monitored and were specifically instructed that no other joint or body motions were permitted that would put tension through the tether connecting the wrist to the force transducer (e.g. no lateral trunk lean, no shoulder shrugging), they could only bend the elbow aiming the thumb towards the ceiling. Because subjects were not stabilized and the limb was not restrained in either fatigue task, the demands placed upon postural stabilizers and accessory muscles, in order to maintain postural alignment and to prevent limb motions in the transverse and frontal planes of motion, were most likely equivalent in this study. Thus, the difference in TTF may be more related to the degree control needed to manage these multiple degrees of freedom within the body. In other words, in addition to the quantity of muscles that needed to be activated for task performance, the two tasks may have required different intracortical control strategies perhaps reflected by differences in the excitability of intracortical circuits to achieve the correct task performance. This type of control would need to be constant throughout task performance, and thus would be independent from the amount and/or rate of change measures of segmental excitability.

The second potential source for the longer duration position-matching task may be due a difference in the sensitivity of or resolution of the visual feedback to detect fluctuations in output. From a motor control perspective, both of these tasks can be considered to be analogous to a sustained visuomotor tracking task because the only way the subject knows that the force output is correct is through constant visual feedback [84]–[86]. From prior studies, the shorter duration task is considered to be the more difficult task by subjects during task performance even though the mechanical demands, torque output, and amount of muscle fatigue at task failure are equivalent between the two tasks [12], [15]. Indeed in this study, subjects anecdotally reported that the force-matching task was “harder” specifically because it was more challenging to keep the feedback line steady. For both tasks, the verbal instructions given to the participant were to “keep the line as close to the target line for as long as you possibly can without using compensations at other joints.” A prior study comparing the effect of visual feedback signal gain during the position-matching task found an increase in TTF for a wider bandwidth of performance [87]. This suggests that had the boundary been wider, performance could have lasted longer. Perhaps it is not just the boundary but also the sensitivity to fluctuations in output that occur within that boundary that matters [85]. The on-screen bandwidth set as the criteria for defining task failure was the same for the two tasks such that the decline in performance output resulted in a similar drop in the net elbow flexor torque for each task. However, the sensitivity of the force transducer to detect fluctuations in force output within that bandwidth and thus project those fluctuations on screen as visual feedback to the subject may have been greater than the sensitivity of the electrogoniometer for detecting the effect of those force fluctuations had on joint angle position. Therefore, during the position-matching task, slight fluctuations in motor output may not be as visually obvious to the participant as compared to the force-matching task and therefore would not merit the need for corrective action by the subject in response to the visual feedback order to maintain their feedback line close to the target line. This suggests that the two tasks may have differed not only in load compliance but also in the demand for feedback-driven corrections in motor output requiring greater cortical control. Together these results, in combination with prior studies about the differences in proximal stabilization and the effect of visual feedback on force control, suggests that there is something more than/other than load compliance that make the TTF for these two tasks differ.

### Supraspinal Contributions to Task Failure

Although there were no task-specific differences found for the changes in excitability in the biceps and for only one variable in the brachioradialis, the data from this experiment adds to the growing understanding of the neurologic mechanisms involved in the decline in force output that limits the duration of a sustained submaximal contraction by investigating the contribution of supraspinal adjustments stemming from changes in intracortical and corticospinal excitability relative to concurrent changes in spinal excitability. At task failure for both the force-matching and position-matching tasks, a similar amount of decline in motorneuron excitability developed as measured by the amplitude of the CMEP elicited in the SP. Additionally, the amount of increase in corticospinal excitability, as measured by the single pulse MEP, was also similar at task failure for both tasks. Therefore, consistent with prior research, both fatigue-tasks ended secondary to a decline in motorneuron excitability coupled with a failure of supraspinal input to successfully sustain motorneuron activity [22], [23], [27]. That task failure occurred for both the force-matching and position-matching tasks after a similar mean decline in motorneuron excitability coupled with a similar mean increase in corticospinal excitability suggests that, in general, the motor cortex is able to compensate for changes in spinal excitability until a particular amount of change develops. At that point, unless more drive is provided from the motor cortex or to the motor cortex, failure occurs. These findings are consistent with the general mechanism for task failure proposed from single motor unit studies, namely task duration is determined by the rate of recruitment of the motor pool by descending drive to compensate for the declining force output from active motor units [12], [17], [22], [23].

The next question to answer then is *why is the motor cortex eventually unable to sustain activation of the motor pool?* To address this question, two competing hypotheses/questions posed in the literature [1], [15], [29] were addressed by the data in this experiment: 1) Is there evidence to support that the cortex itself becomes inhibited, analogous to the spinal cord, which would then decrease the amount of descending drive? 2) Is there evidence to suggest that there is a lack of excitatory drive provided from upstream sources to the motor cortex that would be needed to sustain descending drive?

During the performance of both fatigue-tasks, the duration of the SP increased, which would suggest an increase in intracortical inhibition; however, the direct measures of intracortical excitability do not support this interpretation. First, the amount of intracortical inhibition, as indexed by the value of the SICI ratio, progressively decreased throughout fatigue-task performance to between 147–192% for both tasks in the biceps and to 114–121% in the brachioradialis (although not statistically significant *p* = 0.12). In general, SICI is considered to be mediated locally within the motor cortex such that changes in SICI are interpreted to reflect selective focusing of cortical excitability and corticospinal outputs involved in task performance in the muscle used for voluntary contractions [66], [88]. In addition, prior studies that have used SICI to assess the intracortical changes that develop during fatigue associated with sustained or intermittent MVCs found significant decreases in intracortical inhibition localized to the muscle participating in the task [41]–[43]. Second, the amount of ICF, indexed by the ICF ratio, while greater in the brachioradialis throughout the duration of the force-matching task, the amount of intracortical facilitation did not change during either fatigue-task indicating that there was neither an increase nor decrease in the amount of facilitation within the motor cortex. The ICF ratio, like the SICI ratio, is thought to reflect changes in local motor cortex excitability [20], [37]. One prior study investigating fatigue associated with the performance of intermittent MVCs reported the same result as found in this experiment [42]. Because of the way that that SICI and ICF are evoked through the paired-pulse protocol that investigates the effect of a subthreshold stimulus, that selectively activates the intracortical neurons, on the amplitude of the MEP evoked by the second stimulus, the values for SICI and ICF reflect the excitability of the intracortical circuitry within the motor cortex that synapse upon the corticospinal projections to the motor pool [39], [40], [63], [89]. Therefore, when the normalized values for SICI and ICF were compared to each other, there was a significantly lower amount of intracortical inhibition at task failure for the biceps and no significant change in inhibition in the brachioradialis; however, the amount of intracortical facilitation did not increase as fatigue progressed.

The amount of change that occurred in corticospinal excitability assessed in the presence of voluntary drive, as indexed by the single unconditioned MEP, did not significantly differ from the amount of change in corticospinal excitability measured during the SP when volitional drive was temporarily suspended during both fatigue tasks. Both values increased in both muscles to between 150% and 225% of baseline. If the motor cortex were becoming progressively inhibited as the traditional interpretation of the increased duration of the SP implies, then the amount of change in excitability for the MEP elicited in the SP should not have increased as much as the single MEP evoked in the presence of volitional drive. Additionally, the magnitude of change for the single, unconditioned MEP was not significantly greater than the magnitude of change for the MEP elicited in the SP, which suggests that upstream input to the motor cortex remained consistent relative to the level of ongoing output from the motor cortex as fatigue developed. Further support for this result comes from the lack of change in the value for the LII ratio throughout task performance. This value is the ratio of the two MEP values: the value for the MEP elicited in the SP independent from volitional drive to the value for the MEP evoked in the presence of volitional drive. Therefore, the LII ratio could be considered to represent the effect of volitional drive on corticospinal excitability. Taken together, when compared to the amount of change in the amplitude of the CMEP elicited in the SP (**Figure 10**), these results indicate that increases in corticospinal excitability are not accompanied by active increases in inhibition within the motor cortex. Instead these results indicate that the increase in intracortical excitability that drives the amount of descending drive needed to overcome spinal cord resistance is mediated by decreasing intracortical inhibition that exceeds the amount of change in intracortical facilitation and excitation delivered to the motor cortex from upstream drive. Therefore, the supraspinal mechanisms that limit task duration are most likely mediated by inadequate upstream excitatory drive to the motor cortex, as opposed to increased intracortical inhibition, as the increased SP duration would imply. Upstream drive can be influenced by the level of motivation as reflected by the RPE [1], [9]. It can also be affected by sensory afferents from the periphery to the cortex including both Ia proprioceptive afferents as discussed in the previous section about task differences as well as inputs from the group III and IV metaboreceptors [1], [5], [12]. Recent evidence suggests that the group III and IV afferents have a greater effect on cortical excitability compared to spinal excitability [90]. Finally, when the normalized values for the changes in LII ratio were compared to the normalized SP duration, there was no interaction. Instead, when the normalized SP was compared to the normalized values for the cortical and spinal measures evoked during the SP the results suggest that as the duration of the SP increase, the amount of spinal excitability decreases while the amount of cortical excitability independent from ongoing volitional drive increases. Therefore, these results also support the conclusion that the change in SP duration that occurs during fatiguing contractions is most likely mediated by the progressive decline in spinal excitability and not to an increase in intracortical inhibition as with non-fatiguing contractions.

### Limitations

Although there were no task-specific differences for the total amount of change in spinal and supraspinal excitability that developed over time during fatigue task performance, it should be noted that the effect sizes for some of the pre-fatigue baseline values as well as for two fatigue-task values from the brachioradialis suggest that a main effect for task, independent of contraction duration, may be present. The sample size for this study was calculated from data from previously published studies comparing the time to task failure for the force and position matching tasks and was powered (power = 0.80) to detect significant differences in the time to task failure between the tasks at a *p*<0.05 [17]. Thus, this study was likely underpowered to detect differences under baseline conditions between the two tasks and further work is required to more fully investigate this issue.

## Conclusion

The concurrent use of single pulse, paired-pulse TMS and paired cortio-cervicomedullary stimulation used in this experiment during the performance of the two fatigue tasks (i.e., force-matching and position-matching) provided a unique opportunity to both localize and compare adjustments in segmental excitability in the nervous system to determine the functional significance of the changes to task duration. Although task-specific differences in the neurophysiologic variables were not found in this study, these results do add to a growing body of work that supports the efficacy of the two-task approach to explore the neural mechanisms of task failure. That the opposite task was found to have the shorter TTF (i.e. force-matching <position-matching) in comparison to most reports, supports the conclusion of a task specific difference in central neural command in motor unit recruitment [5], [17], [87]; however, the source of the task specificity may be due to factors other than load compliance such as the amount of stabilization provided to the body and limb and the corrective demand driven by the sensitivity of the visual feedback within the same range of gain [85], [86].

The collective neurophysiologic results across both tasks suggest that, as fatigue develops prior to task failure, the increase in corticospinal excitability observed in relationship to the progressive decline in spinal excitability is the product of decreasing values of intracortical inhibition combined with unchanged values of intracortical facilitation and “upstream” excitability of the motor cortex. Therefore, despite an increase in SP duration, there was no evidence of enhanced intracortical inhibition; instead, these results suggest that the capacity of supraspinal inputs to continue to override spinal resistance is limited by a lack of increase in intracortical facilitation and “upstream” drive to the motor cortex. These results also support the conclusion that during fatiguing submaximal contractions, the increase in SP duration is most likely due to spinal mechanisms reducing motorneuron excitability and therefore, would be best viewed as a measure of composite corticospinal inhibition and not intracortical inhibition as with non-fatiguing contractions [22], [47].

In our subsequent companion paper we present an experiment designed to explore the results presented here that insufficient facilitation and upstream drive to the motor contributes to task failure. Specifically, we address the question of whether anodal transcranial direct current stimulation (tDCS), a non-invasive focal neurostimulation using weak direct electrical currents known to transiently increase cortical excitability, delivered to the motor cortex during the performance of a sustained submaximal contraction increases TTF when compared to a sham tDCS condition.
